# Chemosensory Pathways Involved in the Perception of Tomato-Derived Flavonoids and Alkaloids in *Meloidogyne incognita*

**DOI:** 10.3390/pathogens15080860

**Published:** 2026-08-19

**Authors:** Qian Wang, Liping Peng, Youjing Wang, Jiajia Hu, Liangying Kong, Yajun Liu

**Affiliations:** 1 State Key Laboratory for Conservation and Utilization of Bio-Resources, Yunnan University, Kunming 650091, China; 2 Yunnan Key Laboratory of Basic Research and Innovative Application for Green Biological Production, Yunnan University, Kunming 650091, China

**Keywords:** root-knot nematode, chemotaxis, tomato root exudates, chemosensory genes, RNA interference

## Abstract

Plant-parasitic nematodes rely on the perception of host-derived chemical cues released from plant roots to locate suitable hosts, representing a critical prerequisite for successful plant infection. In this study, we selected four representative tomato (*Solanum lycopersicum* L.) root-derived chemical signals and demonstrated their distinct effects on the host-seeking behavior of the southern root-knot nematode (*Meloidogyne incognita*). Among these compounds, the flavonoids quercetin and luteolin exhibited significant attractant effects, whereas the alkaloids dihydrocapsaicin and solasodine showed repellent effects. RNA interference (RNAi) experiments further indicated that multiple chemosensory neurons are involved in the recognition of plant-derived chemical cues. Silencing of key chemosensory genes significantly impaired the directed chemotactic response of *M. incognita* toward tomato roots and reduced its infectivity, demonstrating that the chemosensory system represents a crucial molecular basis regulating host localization and parasitic establishment. This study provides new insights into host recognition mechanisms in root-knot nematodes and offers a theoretical foundation for developing environmentally sustainable strategies for nematode management by targeting host perception processes.

## 1. Introduction

Plant-parasitic nematodes (PPNs) represent a major group of soil-borne pathogens that pose significant threats to global agricultural production. Among them, root-knot nematodes (*Meloidogyne* spp.) are considered one of the most destructive plant-parasitic nematode groups due to their broad host range, high reproductive capacity, and remarkable environmental adaptability [1]. The *M. incognita* (Kofoid & White) is widely distributed worldwide and infects numerous economically important crops [2]. By inducing the formation of root galls and establishing specialized feeding sites, *M. incognita* disrupts plant water and nutrient acquisition, ultimately resulting in impaired plant growth and substantial yield losses [2]. Current management strategies for root-knot nematodes largely rely on chemical nematicides; however, concerns regarding environmental safety, ecological impacts, and the emergence of nematode resistance have highlighted the urgent need for sustainable and environmentally friendly control approaches [1,3,4].

During the life cycle of root-knot nematodes, the second-stage juvenile (J2) is the only motile and infective stage capable of actively locating and penetrating host roots [5]. After hatching, J2 juveniles must navigate through the soil environment to identify suitable hosts, and successful host localization depends largely on their ability to perceive and respond to chemical signals released from plant roots [6]. Therefore, elucidating how *M. incognita* recognizes plant-derived chemical cues [7] and defining the molecular mechanisms underlying chemotactic behavior are essential for understanding nematode–plant interactions and developing novel behavioral-based intervention strategies.

Root exudates serve as important communication interfaces between plants and the surrounding rhizosphere environment, containing diverse primary and secondary metabolites, including sugars, amino acids, organic acids, phenolic compounds, terpenoids, alkaloids, and flavonoids [8,9]. These compounds not only regulate plant growth and rhizosphere microbial assembly but also function as chemical cues exploited by plant-parasitic nematodes during host searching [10]. Previous studies have demonstrated that root exudates from various crops, including tomato (*Solanum lycopersicum* L.), pepper, and soybean, can induce strong chemotactic responses in root-knot nematodes, whereas certain compounds released by non-host plants exhibit repellent effects [11]. Thus, specific chemical components within plant root exudates may play critical roles in regulating host selection by root-knot nematodes [12]. Previous studies have shown that root-knot nematode infection can alter the composition of tomato root exudates, including changes in flavonoids and alkaloids, such as quercetin and tomatine, which can affect nematode behaviour and development [13,14,15]. In addition, flavonoids, terpenoids, and alkaloids from plants have been reported to exhibit nematicidal, repellent, or behavioural regulatory activities against plant-parasitic nematodes [16,17,18,19,20,21]. Therefore, in this study, candidate natural compounds reported from tomato or other plants with potential effects on root-knot nematodes were collected. Twelve representative compounds belonging to flavonoids/flavonoid derivatives, terpenoids, and alkaloids were initially screened using chemotaxis assays. Based on their stable and significant effects on the chemotactic behaviour of *M. incognita*, quercetin, luteolin, dihydrocapsaicin, and solasodine were selected for subsequent experiments.

Studies using the model nematode *Caenorhabditis elegans* (Maupas) have revealed a highly specialized chemosensory system in nematodes, in which distinct sensory neurons, including AWA, AWC, ASH, and ASE, recognize different classes of different classes of environmental chemical cues [22]. These sensory neurons regulate neuronal activity through conserved signaling modules, including G protein-coupled receptors (GPCRs), receptor-type guanylyl cyclases, cyclic nucleotide-gated (CNG) channels, and transient receptor potential vanilloid (TRPV) channels [23]. Given the similarities in neural organization and conservation of chemosensory-related genes between plant-parasitic nematodes and free-living nematodes, these findings provide important insights into the molecular basis of chemical perception in root-knot nematodes [24]. Previous studies have demonstrated that *M. incognita* homologs of *C. elegans* chemosensory genes contribute to nematode behavioral responses and chemotactic regulation, suggesting that these homologous genes may participate in sensory regulation and chemotactic behavior.

In this study, we investigated the molecular mechanisms underlying chemical perception and host localization in *M. incognita* by integrating plant-derived chemical signal screening, chemotaxis assays, chemosensory gene expression analysis, and RNA interference (RNAi)-mediated functional validation. We aimed to determine how tomato root-derived chemical cues regulate nematode host-seeking behavior and to identify key components of the chemosensory pathway involved in host localization and infection. Our findings provide new insights into the mechanisms underlying host recognition by root-knot nematodes and establish a theoretical foundation for developing environmentally sustainable nematode management strategies based on disruption of host-seeking behavior.

## 2. Materials and Methods

### 2.1. Nematode Materials and Culture Conditions

The *M. incognita* population used in this study was maintained by our research group under laboratory and greenhouse conditions. The population was identified as pure *M. incognita* based on ITS1–5.8S–ITS2 rDNA sequencing and was continuously maintained on tomato plants under greenhouse conditions (25 °C, 50% relative humidity, and a 16 h light/8 h dark photoperiod) as previously described [2]. Second-stage juveniles (J2 juveniles), the infective stage of *M. incognita*, were obtained through egg mass hatching. The freshly hatched J2s were rinsed with sterile water and subsequently used for chemotaxis assays, gene expression analysis, RNA interference (RNAi) experiments, and host localization assays [9].

### 2.2. Screening of Plant-Derived Chemical Signals and Agar Plate Chemotaxis Assays

Different concentrations of each compound were initially tested to determine the concentration-dependent effects on the chemotactic behaviour of *M. incognita*. The concentration showing the strongest and most stable attractive or repellent response was selected as the optimal concentration for subsequent RNA interference and phenotypic validation experiments. The selected compounds included the flavonoids quercetin (10^−3^ mg/mL) (Solarbio, Beijing, China) and luteolin (10^−4^ mg/mL) (Aladdin, Shanghai, China), as well as the alkaloids dihydrocapsaicin (10^−4^ mg/mL) (Aladdin, Shanghai, China) and solasodine (10^−4^ mg/mL) (Solarbio, Beijing, China).

An agar plate chemotaxis assay was performed to evaluate the behavioral responses of *M. incognita* J2 juveniles toward different plant-derived chemical signals. Chemotaxis plates were prepared using 1% agarose, with the compound treatment zone and solvent control zone positioned on opposite sides of the plate. Approximately 200 J2 juveniles were placed in the center of each plate and allowed to migrate at 28 °C in darkness for 2 h. After incubation, the number of nematodes in the compound and control regions was recorded, and the chemotaxis index (*CI*) was calculated according to the following formula:

$$CI=\frac{N_{c}-N_{s}}{N_{c}+N_{s}}$$

where *N_c_* represents the number of nematodes in the chemical compound region and *N_s_* represents the number of nematodes in the solvent control region. A *CI* value between −0.1 and 0.1 was considered to indicate no significant response, whereas *CI* > 0.1 and *CI* < −0.1 represented attraction and repulsion responses, respectively [6]. Each treatment consisted of three independent biological replicates, with freshly hatched J2 juveniles prepared independently for each replicate. The chemotaxis assay was performed using independent nematode preparations, and the mean value from each biological replicate was used for statistical analysis.

### 2.3. Identification and Expression Analysis of Candidate Chemosensory Genes

Candidate chemosensory genes were selected based on previous studies of the chemosensory systems of *C. elegans* and *M. incognita*, combined with the structural characteristics and reported biological functions of the selected plant-derived compounds. Candidate genes mainly included homologs involved in the AWA, AWC, ASH, and ASE chemosensory pathways.

Protein sequences of candidate genes were obtained from the *C. elegans* database [25] and subjected to BLAST analysis using the NCBI BLAST online tool (https://blast.ncbi.nlm.nih.gov/Blast.cgi, accessed in 16 August 2026) homology searches against the *M. incognita* genome database [26] to identify highly homologous genes. The homologous relationships between *M. incognita* genes and their *C. elegans* counterparts are summarized in Table S1. Conserved functional domains were further analyzed using the NCBI database. Gene-specific primers were designed using the NCBI Primer-BLAST online tool, with expected amplicon sizes ranging from 80 to 150 bp; specific primers used for RT-qPCR analysis are listed in Table S2. Primer specificity was further verified by BLAST analysis against the *M. incognita* (Kofoid & White) genome database.

Following the chemotaxis assay, J2 juveniles located in the chemical treatment region and solvent control region were separately collected, immediately frozen in liquid nitrogen, and stored at −80 °C for subsequent analysis. Total RNA was extracted using the RaPure Total RNA Micro Kit (Magen, Shanghai, China), and first-strand cDNA was synthesized using the PrimeScript™ RT reagent Kit with gDNA Eraser (TaKaRa, Beijing, China). Quantitative real-time PCR (RT-qPCR) was performed using TB Green^®^ Premix Ex Taq™ II (TaKaRa, Beijing, China). The amplification program consisted of an initial denaturation at 95 °C for 30 s, followed by 40 cycles of 95 °C for 5 s, 55 °C for 30 s, and 72 °C for 30 s. Melting curve analysis was conducted to verify amplification specificity. The *β-actin* gene was used as the internal reference, and relative gene expression levels were calculated using the 2^−ΔΔCt^ method. Each treatment contained three independent biological replicates. For each biological replicate, three technical replicates were performed, and the average Ct value was used for relative expression analysis.

### 2.4. Cloning of Candidate Chemosensory Genes and Synthesis of dsRNA

Specific primers were designed according to candidate gene sequences to amplify target fragments of approximately 450–600 bp. A T7 promoter sequence was incorporated into the 5′ end of each primer for subsequent in vitro transcription. The primer sequences used for dsRNA synthesis are provided in Table S3. Total RNA extraction and cDNA synthesis were performed as described in Section 2.3.

PCR-amplified products were purified and cloned into the pMD19-T Simple Vector (TaKaRa, Beijing, China), followed by transformation into *Escherichia coli* DH5α competent cells. Recombinant plasmids were verified by colony PCR and sequencing. The recombinant plasmids containing the correct inserts were used as templates to amplify target fragments containing T7 promoter sequences. Double-stranded RNA (dsRNA) was synthesized using the T7 High Yield RNA Transcription Kit (Vazyme, Nanjing, China) and purified using the MagBeads RNA Purification Kit (MP Biomedicals, Shanghai, China). The concentration and integrity of purified dsRNA were determined by nucleic acid quantification and agarose gel electrophoresis. A non-specific dsRNA sequence (nc-dsRNA) was used as the negative control.

### 2.5. RNA Interference and Functional Analysis

RNA interference assays were performed using an in vitro soaking method. Approximately 20,000 *M. incognita* J2 juveniles were incubated in 500 μL RNAi solution containing 10 μL of 5% resorcinol, 50 μL of 30 μM spermidine, 5 μL of 5% gelatin, dsRNA, and M9 buffer to a final volume of 500 μL. The final dsRNA concentration ranged from 100 to 400 ng/μL. Nematodes were incubated at 28 °C in darkness for 24 h, washed with RNase-free water, and allowed to recover for an additional 24 h. Buffer-treated and nc-dsRNA-treated nematodes were used as controls.

The silencing efficiency of each target gene was evaluated by RT-qPCR using three independent biological replicates. For each biological replicate, three technical replicates were conducted, and the average value was used for statistical analysis. Following RNAi treatment and recovery, locomotion ability of J2 juveniles was evaluated. Equal volumes of nematode suspensions were placed on microscope slides, and actively moving nematodes were counted under a stereomicroscope. Locomotion ability was expressed as the percentage of actively moving nematodes relative to the total number of nematodes.

The chemotactic response of RNAi-treated nematodes toward tomato roots was evaluated using a gel-based root chemotaxis system. A 23% Pluronic F-127 gel solution was prepared and maintained at 4 °C until complete dissolution. Tomato seeds (cv. 903) were surface sterilized with 1% sodium hypochlorite (NaClO) for 10 min, rinsed five times with sterile distilled water, and germinated on moist filter paper at 28 °C in darkness for 72 h. Seedlings with approximately 2 cm root length were used for chemotaxis assays. Two milliliters of gel solution were evenly distributed into 3.5 cm Petri dishes and allowed to solidify. Tomato seedlings were positioned on one side of the plate, and approximately 200 RNAi-treated J2 juveniles were introduced into the nematode inoculation zone. Plates were incubated at 28 °C in darkness, and the number of nematodes accumulated around tomato roots was recorded at 4, 8, 12, and 24 h. Each treatment consisted of three independent biological replicates, with approximately 200 RNAi-treated J2 juveniles used for each replicate.

### 2.6. Evaluation of Infectivity of RNAi-Treated M. incognita on Tomato Roots

Twenty-four hours after the nematode chemotaxis assay toward tomato roots, the infectivity of RNAi-treated *M. incognita* J2 juveniles was evaluated. Tomato roots were stained using an acid fuchsin staining method following treatment with 1.0% sodium hypochlorite. After staining, roots were cleared with a 1:1 mixture of lactic acid and glycerol. The number of nematodes penetrated into root tissues was observed and counted under a microscope to assess the infection ability of RNAi-treated nematodes. Each treatment consisted of three independent biological replicates, and each replicate represented an independent infection assay using independently prepared RNAi-treated nematodes.

### 2.7. Statistical Analysis

Statistical analyses were performed using GraphPad Prism 10.0. Data are presented as mean ± SD from three independent biological replicates (*n* = 3). For RT-qPCR experiments, three technical replicates were performed for each biological replicate, and the average value was used for statistical analysis. Before ANOVA, data were evaluated for normality using the Shapiro–Wilk test and for homogeneity of variance using the Brown–Forsythe test. All datasets met the assumptions required for ANOVA (*p* > 0.05). Statistical significance was determined using two-way ANOVA followed by Tukey’s multiple comparison test. Differences were considered statistically significant at *p* < 0.05.

## 3. Results

### 3.1. Plant-Derived Chemical Signals Induce Differential Chemotactic Responses in M. incognita

To screen plant-derived chemical signals that regulate the behavioral responses of *M. incognita*, representative secondary metabolites, including flavonoids and alkaloids, were selected as candidate plant-derived chemical cues based on previous metabolomic analyses and reported studies. The effects of these compounds on the behavioral preference of second-stage juveniles (J2) of *M. incognita* were evaluated using an agarose plate chemotaxis assay.

**Figure 1:**
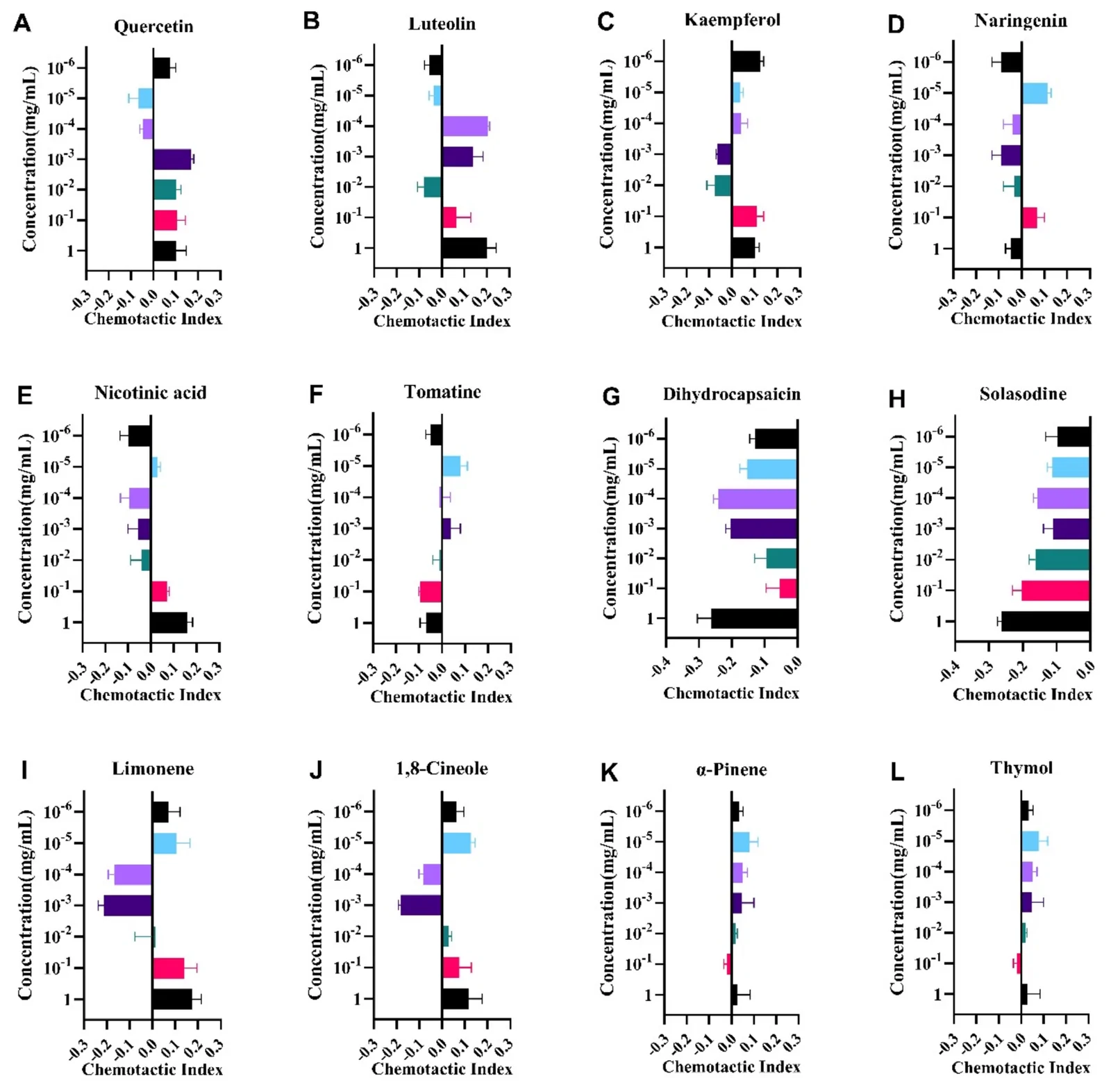
Chemotactic responses of *M. incognita* second-stage juveniles (J2s) to different concentrations of plant-derived compounds. (**A**) Quercetin; (**B**) luteolin; (**C**) kaempferol; (**D**) naringenin; (**E**) nicotinic acid; (**F**) tomatine; (**G**) dihydrocapsaicin; (**H**) solasodine; (**I**) limonene; (**J**) 1,8-cineole; (**K**) α-pinene; and (**L**) thymol. The chemotaxis index (*CI*) indicates the behavioral response of *M. incognita* J2s toward different concentrations of plant-derived compounds, with positive and negative values representing attraction and repulsion, respectively. *CI* values between −0.1 and 0.1 were considered as no significant behavioral response. Data are presented as mean ± SD from three independent biological replicates (*n* = 3).

The results showed that different plant-derived compounds induced distinct chemotactic responses in *M. incognita*. Among the tested flavonoids, several compounds triggered positive chemotactic responses. Quercetin and luteolin exhibited strong attractant effects on J2 juveniles (Figure 1A,B), whereas kaempferol and naringenin induced relatively weak positive responses (Figure 1C,D). In contrast to flavonoids, the tested alkaloids primarily induced avoidance behaviors. Treatment with dihydrocapsaicin and solasodine resulted in consistent negative chemotactic responses in *M. incognita* (Figure 1G,H), while nicotinic acid and tomatine exhibited weaker repellent effects (Figure 1E,F).

To further evaluate the effects of volatile terpenoid compounds on the behavioral responses of *M. incognita*, four representative terpenoids, including thymol, limonene, 1,8-cineole, and α-pinene, were selected for chemotaxis analysis. The results showed that none of the tested terpenoids induced a persistent and significant chemotactic preference in J2 juveniles. Although slight attraction or repulsion responses were observed at certain concentrations, these responses were generally weak and showed concentration-dependent variation (Figure 1I–L). Compared with the tested flavonoids and alkaloids, these terpenoid compounds exhibited limited effects on the behavioral regulation of *M. incognita*.

Collectively, behavioral screening of different classes of plant-derived secondary metabolites identified four representative chemical signals with distinct chemotactic phenotypes, including the attractant signals quercetin and luteolin, as well as the repellent signals dihydrocapsaicin and solasodine. These findings established an association between plant-derived chemical signals and behavioral responses of *M. incognita*, providing an experimental foundation for further investigation of the molecular mechanisms underlying chemosensory perception.

### 3.2. Plant-Derived Chemical Signals Induce Differential Expression of Chemosensory-Related Genes in M. incognita

To investigate the molecular mechanisms underlying the perception of plant-derived chemical signals in *M. incognita*, candidate chemosensory-related genes were selected based on the reported chemosensory neuronal system of *C. elegans* and homologous gene information from *M. incognita*. The selected genes were mainly involved in pathways associated with AWA, AWC, ASH, and ASE chemosensory neurons [27]. A total of 36 homologous genes involved in chemical signal recognition were selected for expression analysis.

**Figure 2:**
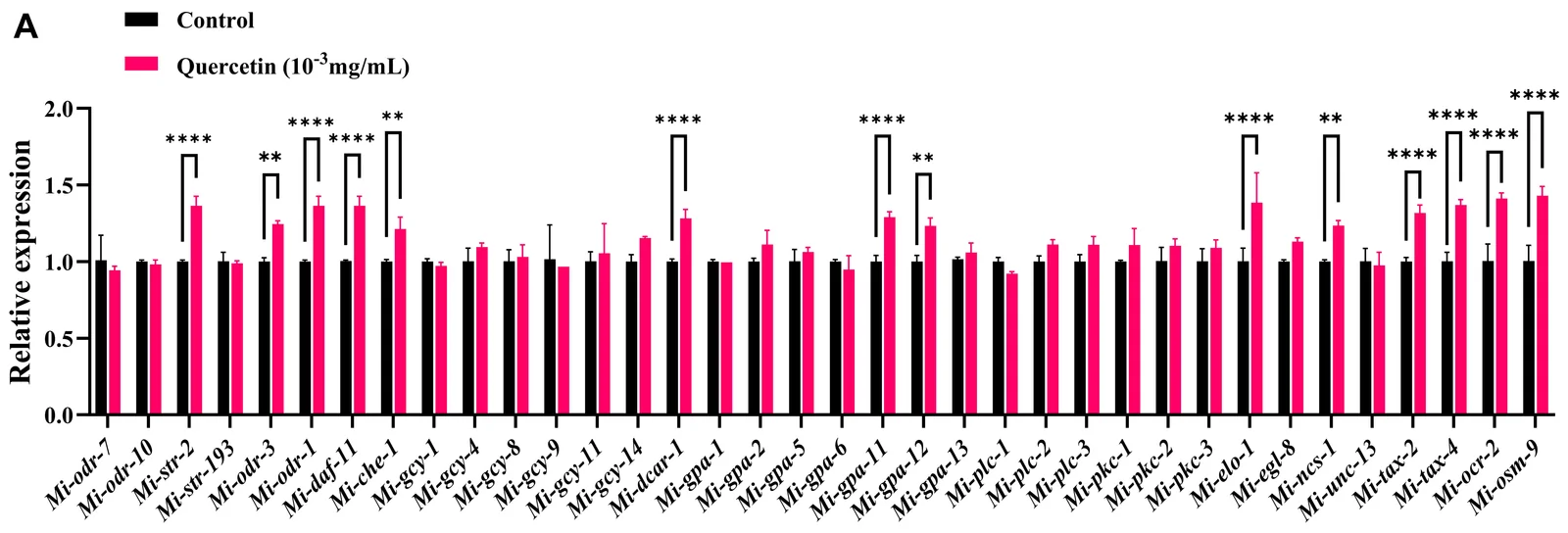

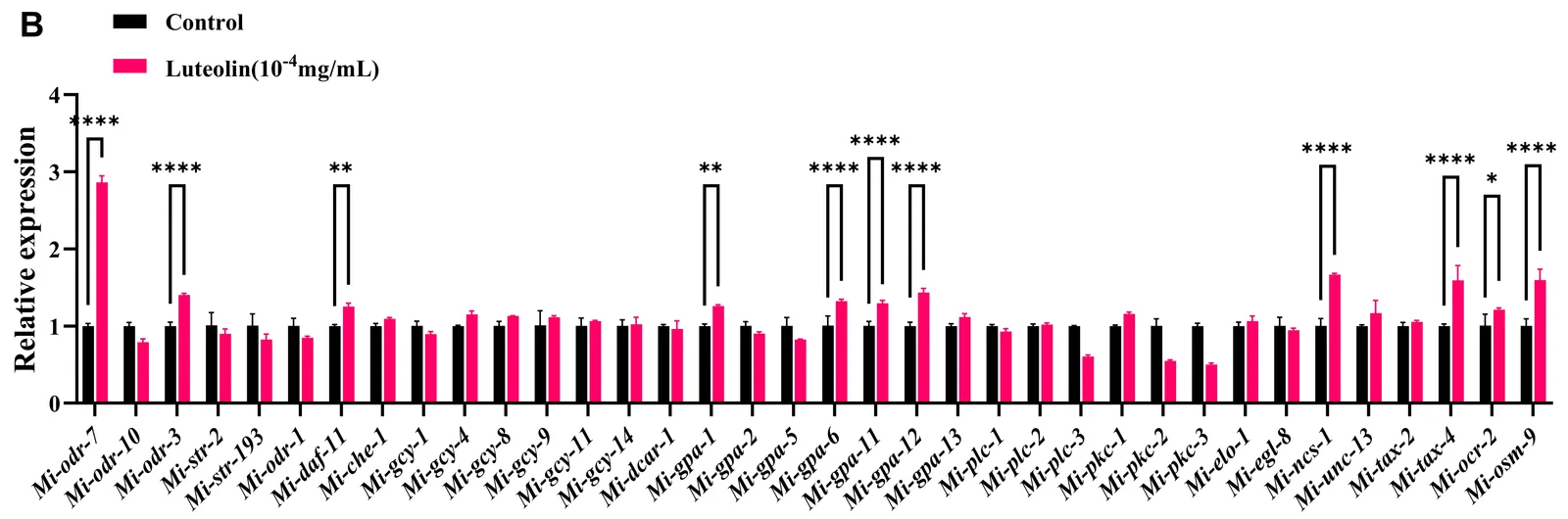

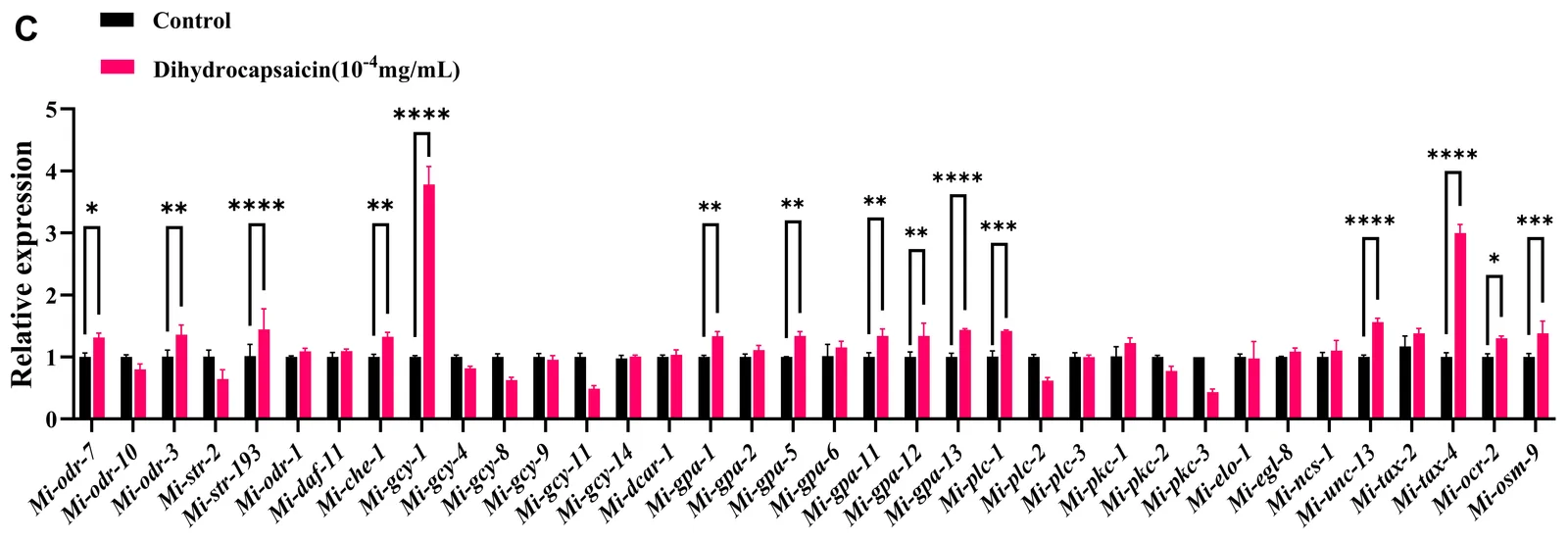

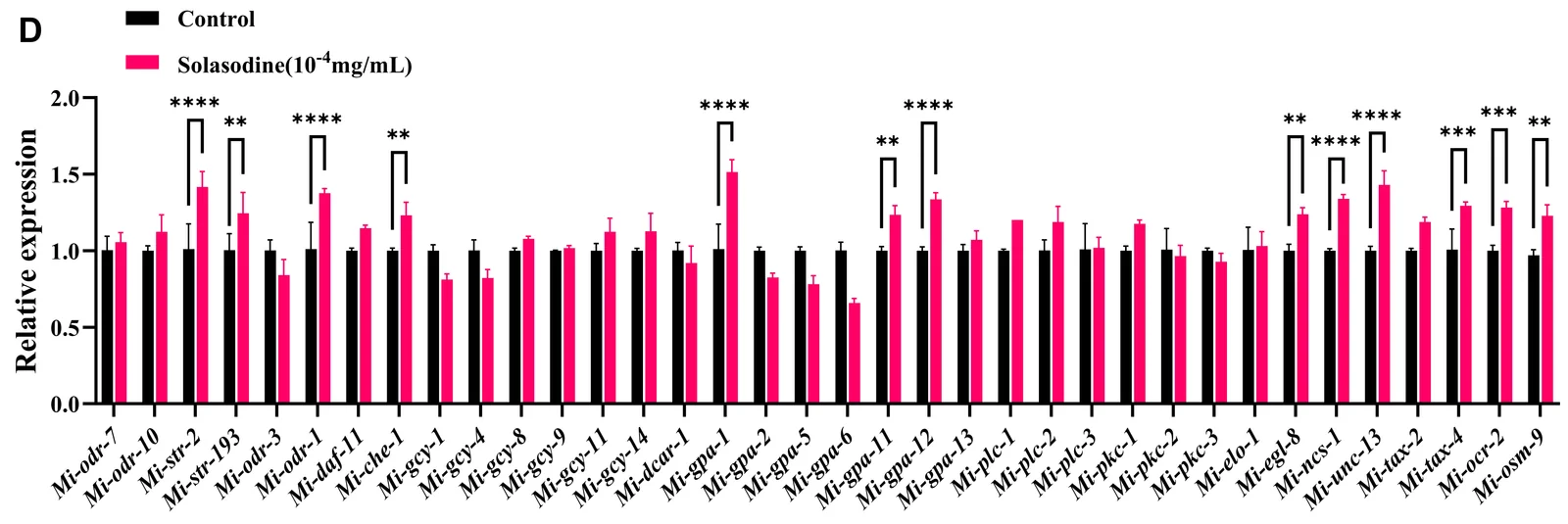
Plant-derived chemical signals induce differential expression of chemosensory-related genes in *M. incognita* J2s. (**A**) Quercetin; (**B**) luteolin; (**C**) dihydrocapsaicin; and (**D**) solasodine. Relative transcript levels of candidate chemosensory-related genes were determined by RT-qPCR. Data are presented as mean ± SD from three independent biological replicates (*n* = 3). For each biological replicate, three technical replicates were performed, and the average value was used for statistical analysis. Statistical significance was determined using two-way ANOVA followed by Tukey’s multiple comparison test. * *p* < 0.05, ** *p* < 0.01, *** *p* < 0.001, and **** *p* < 0.0001 were considered statistically significant.

Treatment with attractant compounds induced significant transcriptional changes in multiple chemosensory-related genes. Quercetin treatment significantly increased the expression levels of 14 candidate genes (Figure 2A), whereas luteolin treatment induced significant upregulation of 11 genes (Figure 2B). Although both compounds belong to the flavonoid family, the responsive gene profiles showed distinct patterns between quercetin and luteolin treatments, suggesting that structurally related flavonoids may trigger distinct chemosensory responses in *M. incognita*.

Repellent compounds also triggered differential expression of chemosensory-related genes. Dihydrocapsaicin treatment resulted in significant transcriptional upregulation of 15 candidate genes (Figure 2C), while solasodine treatment induced increased expression of 13 genes (Figure 2D). Compared with attractant compounds, the responsive gene sets induced by repellent signals displayed distinct expression patterns, suggesting that *M. incognita* may employ specific molecular recognition mechanisms for the perception of different types of environmental chemical cues.

### 3.3. RNA Interference-Mediated Silencing of Candidate Genes Involved in Plant-Derived Chemical Signal Responses

To further investigate the potential functions of candidate genes involved in the perception of plant-derived chemical signals, representative genes were selected for RNA interference (RNAi)-based functional validation according to their compound-induced expression patterns, associated sensory neuron types, and previously reported functional information. The expression levels of target genes after RNAi treatment were analyzed by RT-qPCR to evaluate the efficiency of gene silencing.

**Figure 3:**
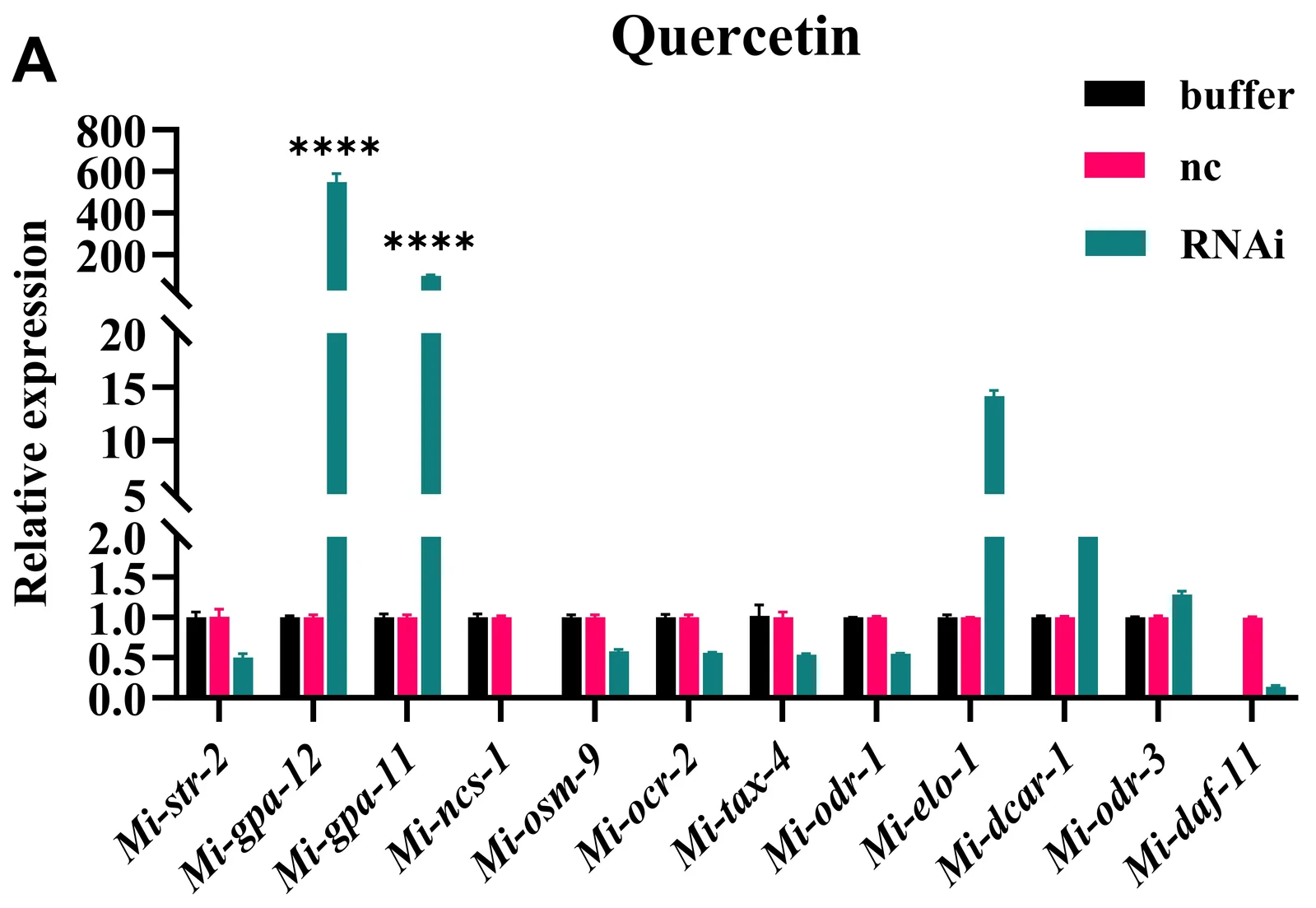

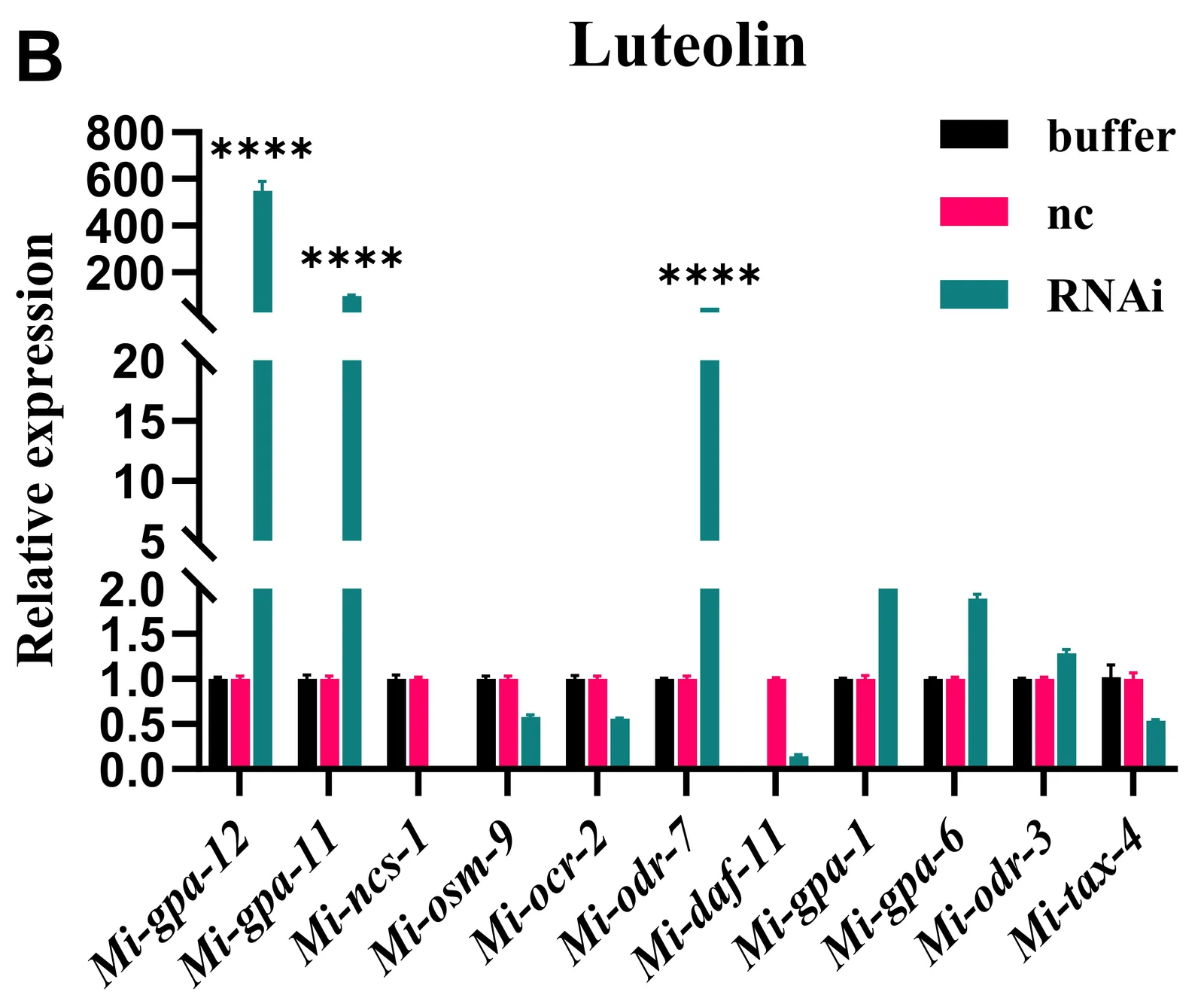

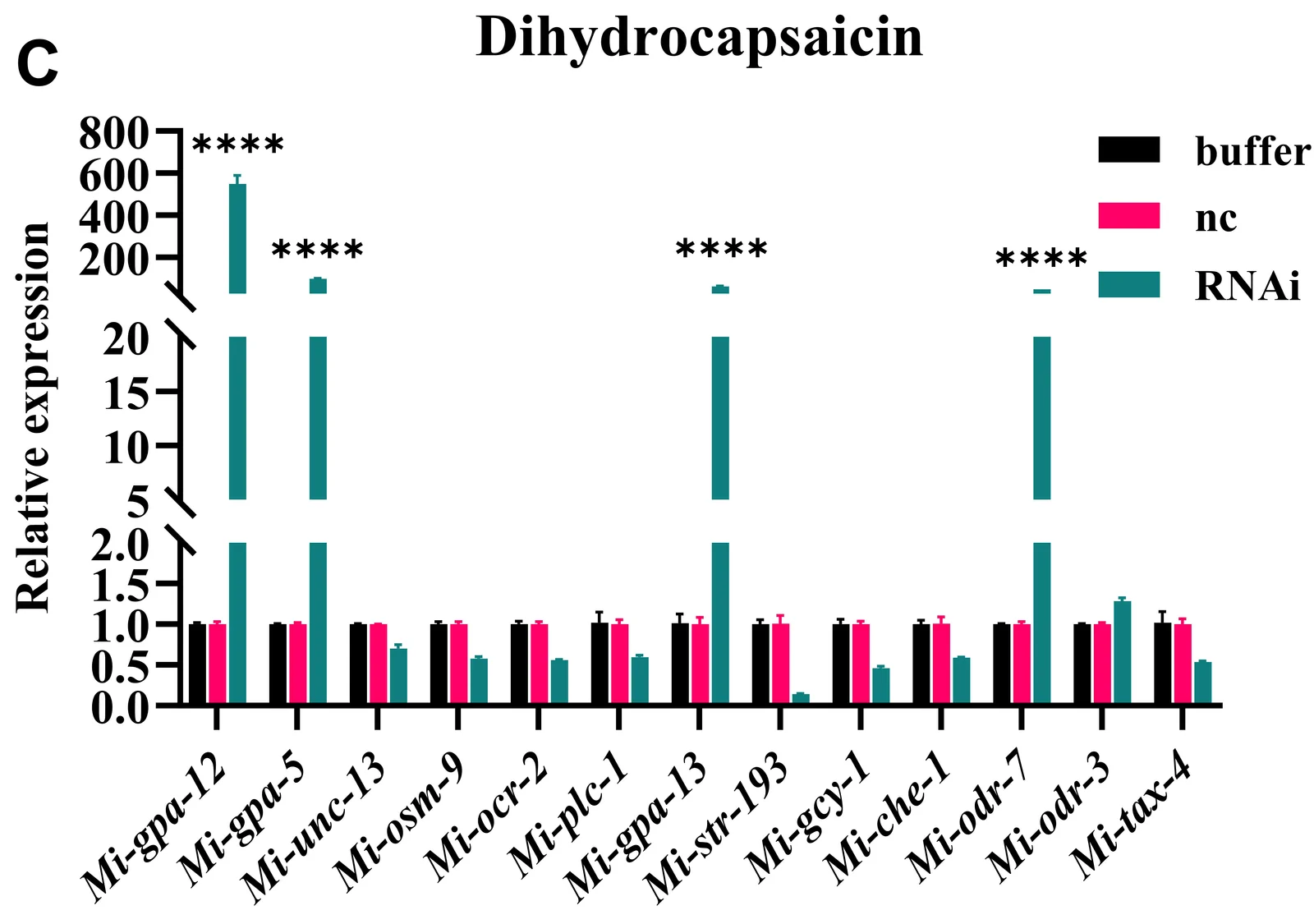

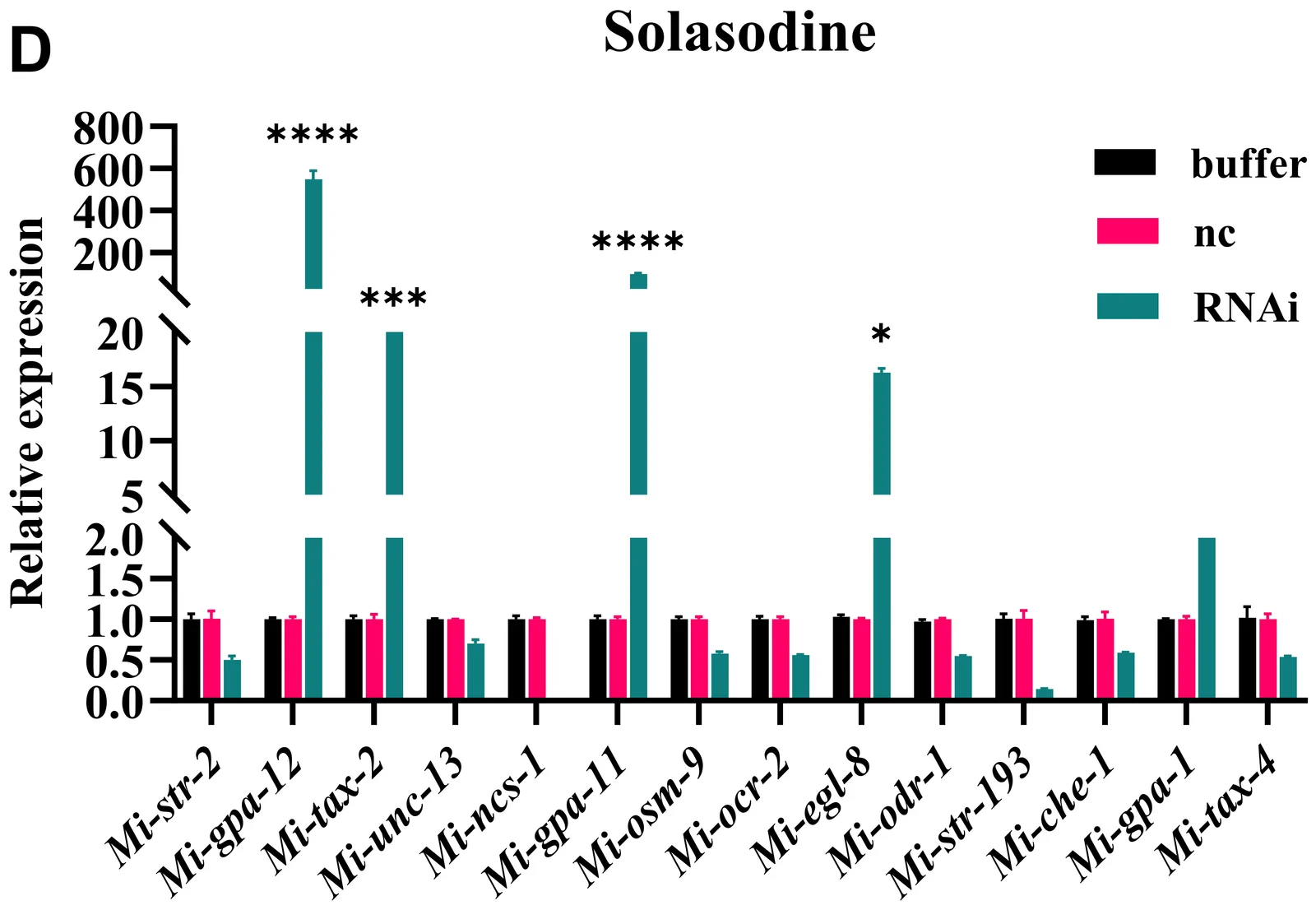
Expression responses of candidate chemosensory genes in *M. incognita* following RNA interference treatment. (**A**) Quercetin-responsive genes; (**B**) luteolin-responsive genes; (**C**) dihydrocapsaicin-responsive genes; (**D**) solasodine-responsive genes. Relative gene expression levels were calculated using the 2^−ΔΔCt^ method with *β-actin* as the reference gene. Data are presented as mean ± SD from three independent biological replicates (*n* = 3). Asterisks indicate significant differences compared with the nc-dsRNA control group. Statistical significance was determined using two-way ANOVA followed by Tukey’s multiple comparison test. * *p* < 0.05, *** *p* < 0.001, and **** *p* < 0.0001 were considered statistically significant.

Compared with the buffer-treated and nc-dsRNA control groups, candidate genes associated with different plant-derived chemical signals exhibited varying degrees of transcriptional responses following RNAi treatment (Figure 3). In the quercetin-responsive gene silencing experiment (Figure 3A), the expression levels of most target genes were significantly reduced after dsRNA treatment, with several genes showing pronounced downregulation, indicating that the corresponding dsRNAs effectively reduced target gene transcript abundance. Similarly, in the luteolin-responsive gene RNAi treatment (Figure 3B), the selected candidate genes exhibited different levels of silencing efficiency, and the transcript abundance of most genes was significantly decreased compared with the nc-dsRNA control group.

For genes responsive to avoidance-inducing chemical signals, RNAi treatment targeting dihydrocapsaicin-responsive and solasodine-responsive genes resulted in reduced transcript levels for several candidates, although the efficiency of gene suppression varied among different targets (Figure 3C,D). These results indicate that individual candidate genes may differ in their sensitivity to RNAi-mediated regulation. Overall, the candidate chemosensory genes identified in response to different plant-derived chemical signals could be effectively regulated through dsRNA soaking-mediated RNAi. However, several genes, including *Mi-gpa-12*, *Mi-gpa-11*, and *Mi-odr-7*, exhibited increased transcript abundance following dsRNA treatment, suggesting that different chemosensory genes may undergo distinct transcriptional responses after RNAi exposure. These results provide a foundation for further investigating the roles of key chemosensory genes in regulating chemical-directed behavior and host localization in *M. incognita*.

### 3.4. RNA Interference Alters the Chemotactic Responses of M. incognita to Plant-Derived Chemical Signals

To further verify the roles of candidate chemosensory genes in regulating behavioral responses to plant-derived chemical signals, RNA interference (RNAi)-treated *M. incognita* J2 juveniles were subjected to chemotaxis assays using representative attractant and repellent compounds.

**Figure 4:**
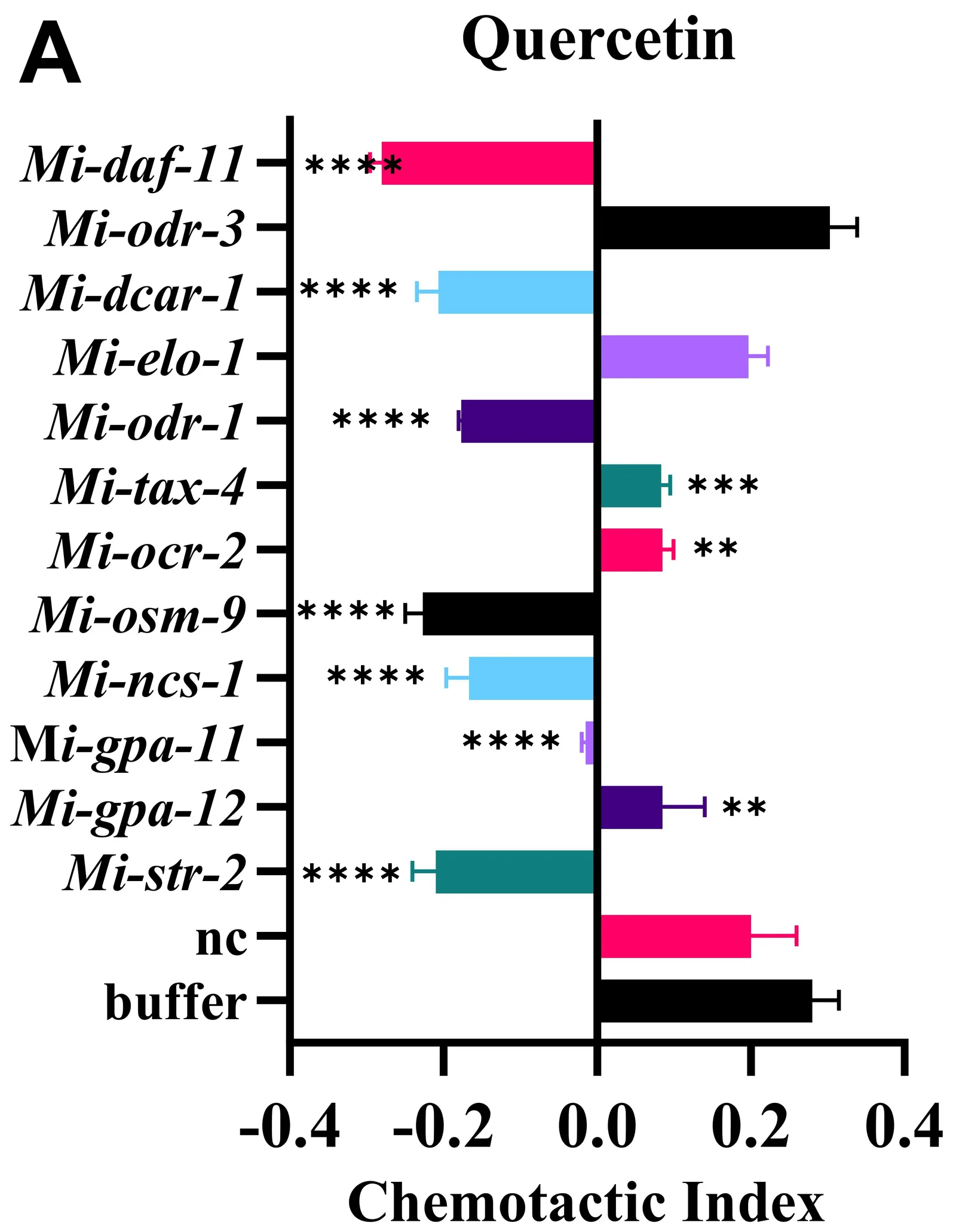

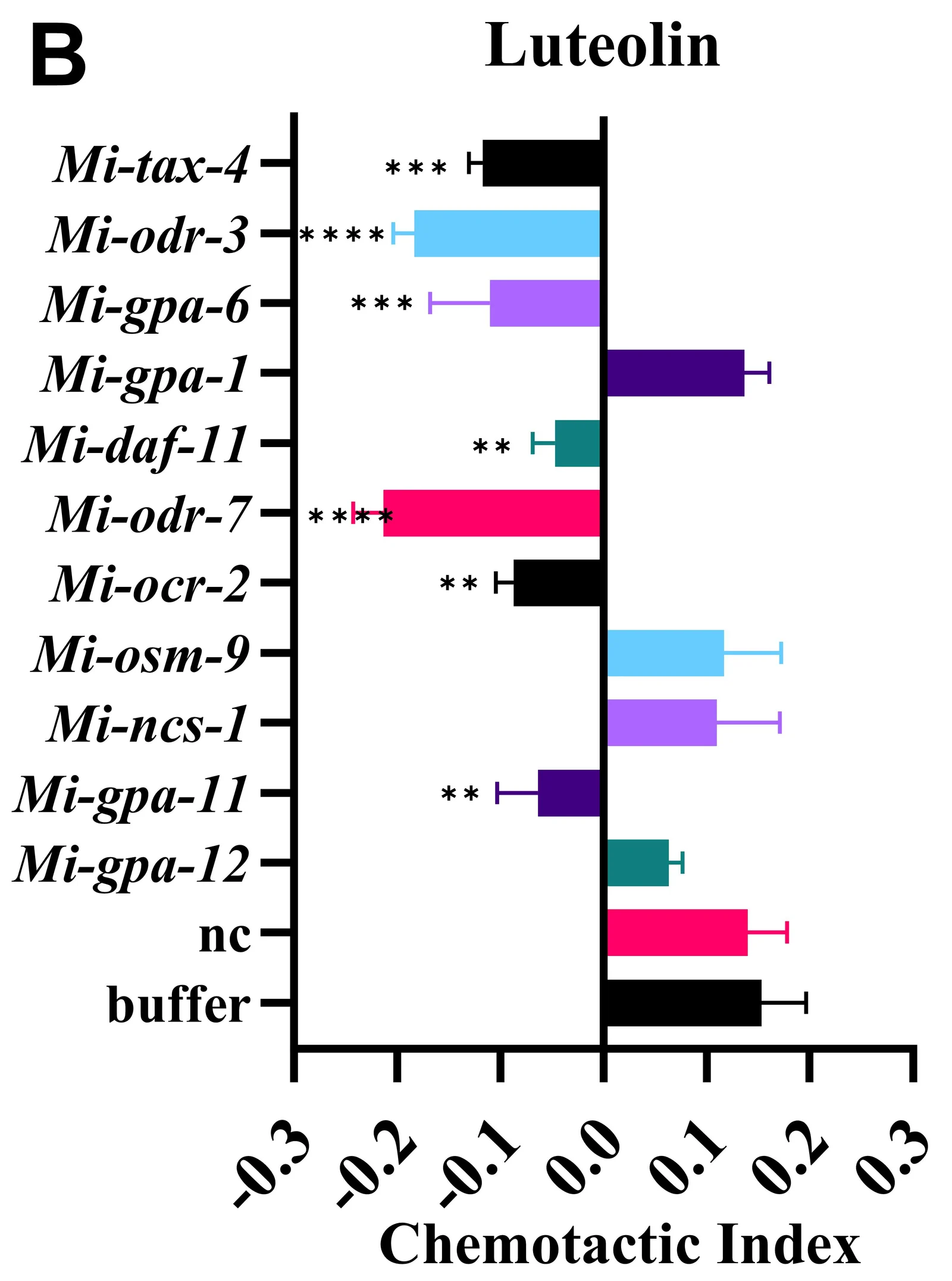

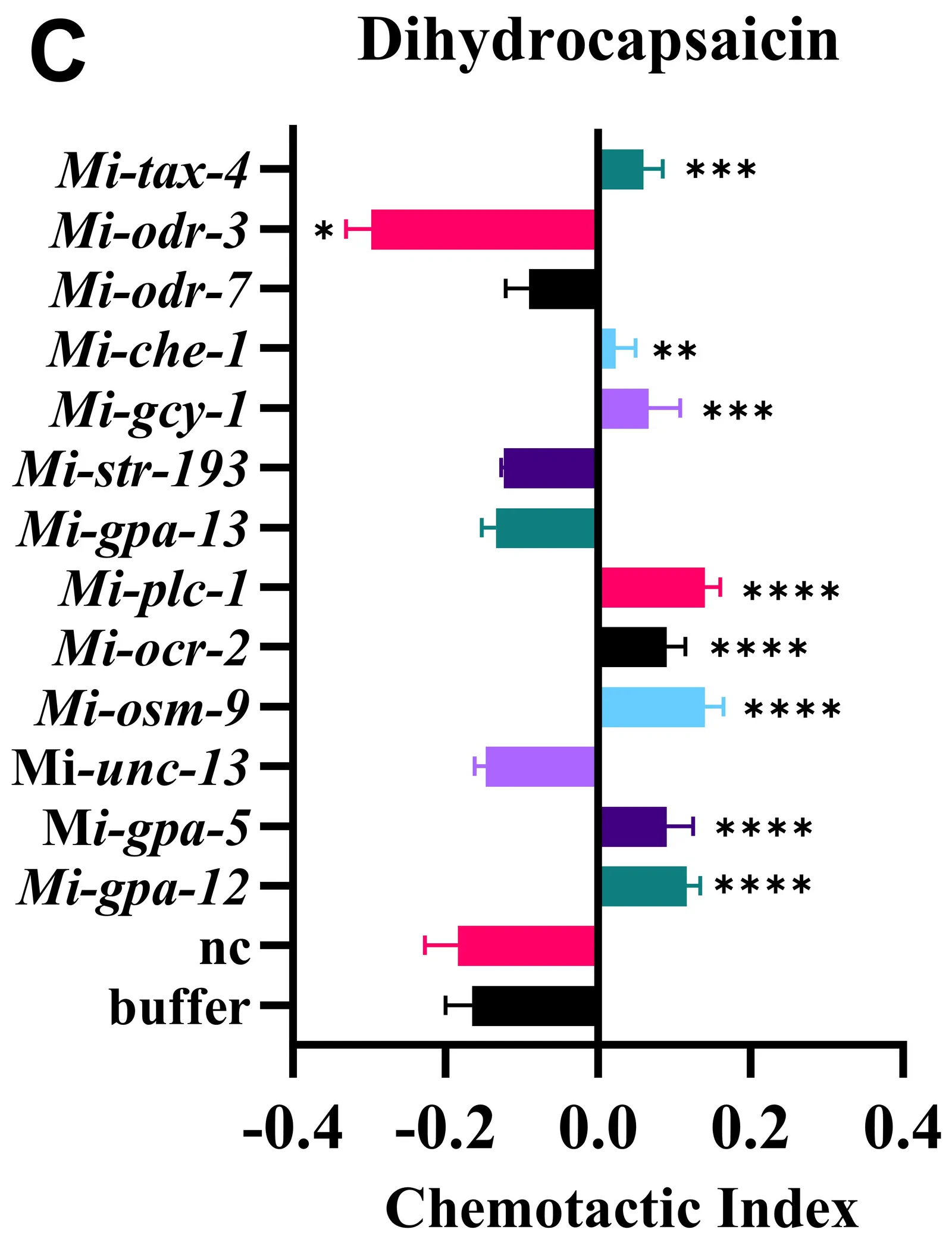

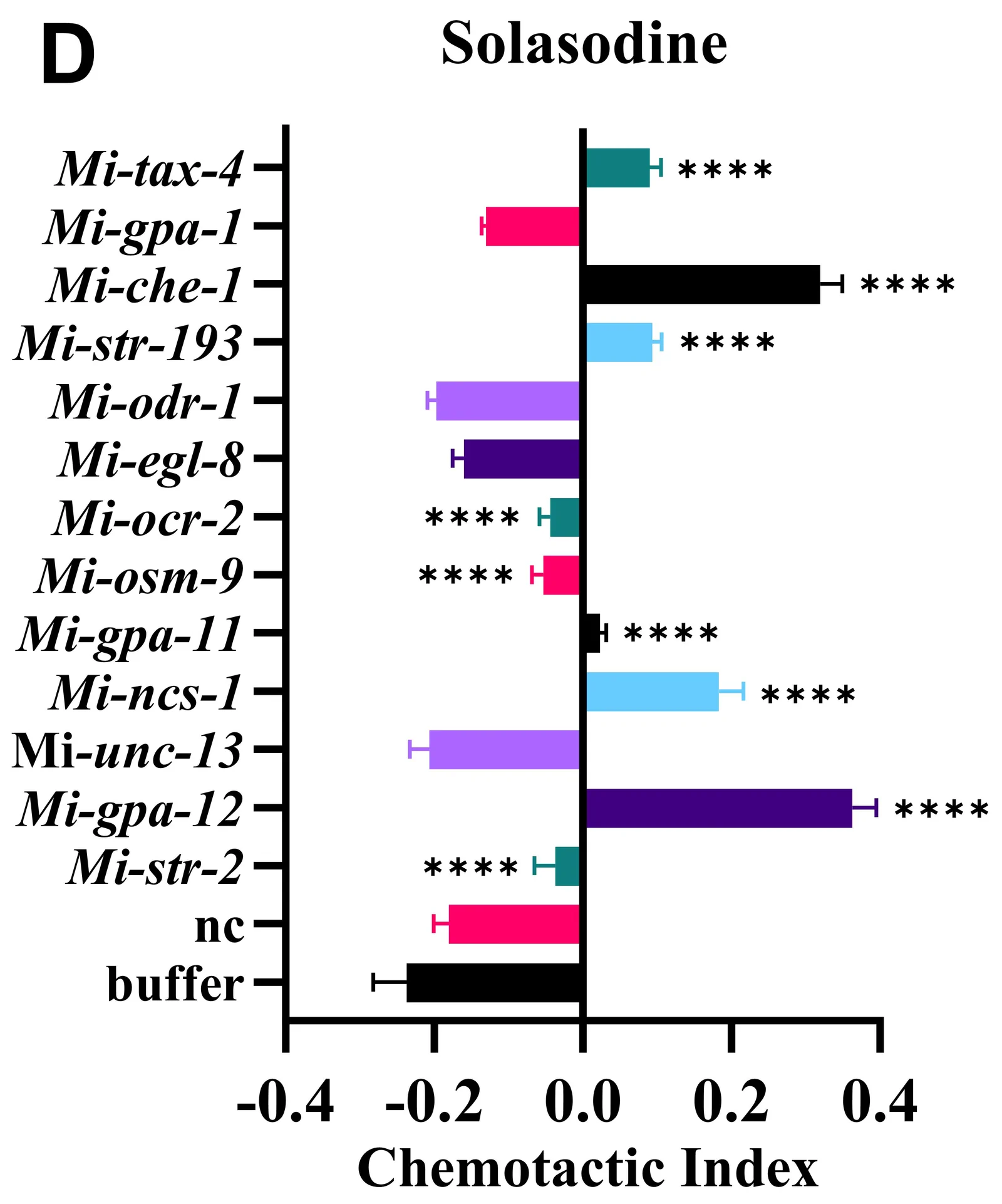
Effects of RNA interference on the chemotactic responses of *M. incognita* J2s to plant-derived chemical signals. (**A**–**D**) represent responses to quercetin, luteolin, dihydrocapsaicin, and solasodine, respectively. Bars represent the chemotaxis index (*CI*) of different RNAi-treated groups in response to each chemical signal. Data are presented as mean ± SD from three independent biological replicates (*n* = 3). Asterisks indicate significant differences compared with the nc-dsRNA-treated control group. Statistical significance was determined using one-way ANOVA followed by Tukey’s multiple comparison test. * *p* < 0.05, ** *p* < 0.01, *** *p* < 0.001, and **** *p* < 0.0001 were considered statistically significant.

The results showed that silencing of candidate chemosensory genes differentially affected the behavioral responses of *M. incognita* to plant-derived chemical signals. Under quercetin treatment, knockdown of several candidate genes significantly altered the chemotactic response of J2 juveniles. Specifically, silencing of *Mi-dcar-1*, *Mi-odr-1*, *Mi-osm-9*, *Mi-ncs-1*, and *Mi-str-2* significantly reduced chemotactic attraction toward quercetin, whereas knockdown of other genes did not result in significant changes in chemotaxis (Figure 4A). These results indicated that specific chemosensory-related genes contribute to the perception and behavioral response of *M. incognita* to quercetin.

Similarly, RNAi-mediated gene silencing affected the chemotactic response toward luteolin. Knockdown of *Mi-odr-7*, *Mi-gpa-6*, *Mi-odr-3*, and *Mi-tax-4* significantly decreased the chemotaxis index of J2 juveniles toward luteolin, whereas silencing of other candidate genes did not significantly affect the response (Figure 4B). These findings suggested that distinct chemosensory components are involved in the perception of different flavonoid-derived attractant signals.

For the repellent compound dihydrocapsaicin, RNAi treatment also modified the avoidance behavior of *M. incognita*. Silencing of *Mi-plc-1*, *Mi-ocr-2*, *Mi-osm-9*, *Mi-gpa-5*, and *Mi-gpa-12* significantly reduced the negative chemotactic response toward dihydrocapsaicin, indicating that these genes are involved in the perception of this repellent signal (Figure 4C). In contrast, *Mi-odr-3* silencing did not eliminate the repellent response, as RNAi-treated juveniles maintained a strong negative chemotactic preference, suggesting that other chemosensory components may compensate for its loss of function.

Under solasodine treatment, silencing of candidate chemosensory genes similarly influenced the repellent behavior of *M. incognita*. Knockdown of *Mi-tax-4*, *Mi-che-1*, *Mi-str-193*, *Mi-gpa-11*, *Mi-ncs-1*, and *Mi-gpa-12* significantly weakened the negative chemotactic response toward solasodine (Figure 4D). However, silencing of several other genes did not significantly affect avoidance behavior, further demonstrating that different chemosensory genes exhibit compound-specific functional contributions during the recognition of plant-derived chemical signals.

Collectively, RNAi-mediated silencing of candidate chemosensory genes altered the behavioral preferences of *M. incognita* toward different plant-derived chemical signals. The reduced attraction or repulsion responses observed after specific gene knockdown indicated that these genes contribute to the perception of plant-derived signals and host localization behavior in *M. incognita*.

### 3.5. RNA Interference Targeting Chemosensory Genes Does Not Affect the Locomotor Ability of M. incognita

To exclude the possibility that impaired locomotion after RNAi treatment contributed to altered chemotactic responses, the motility of RNAi-treated *M. incognita* J2 was evaluated by quantifying the number of migrating nematodes within a defined time period.

**Figure 5:**
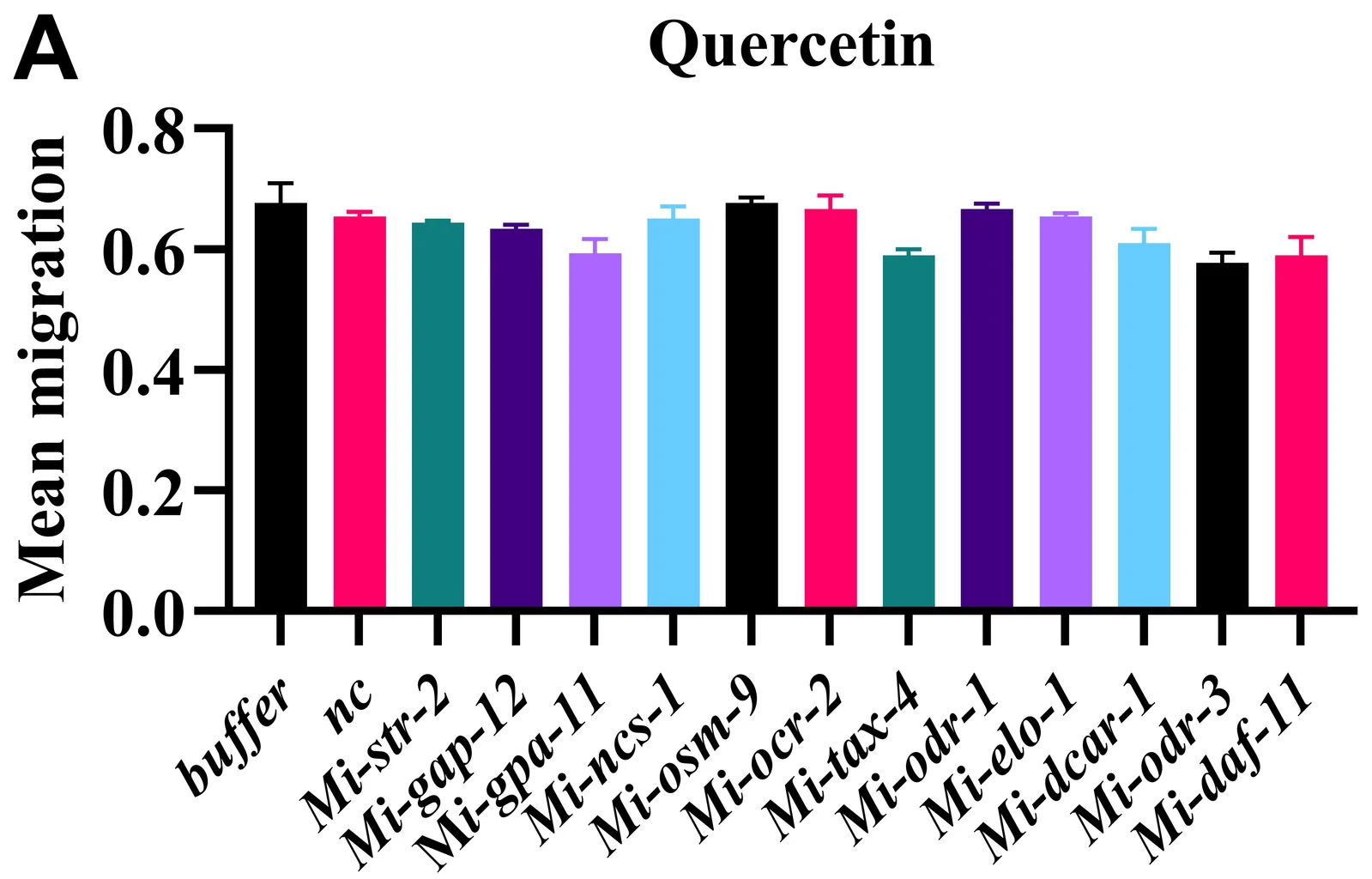

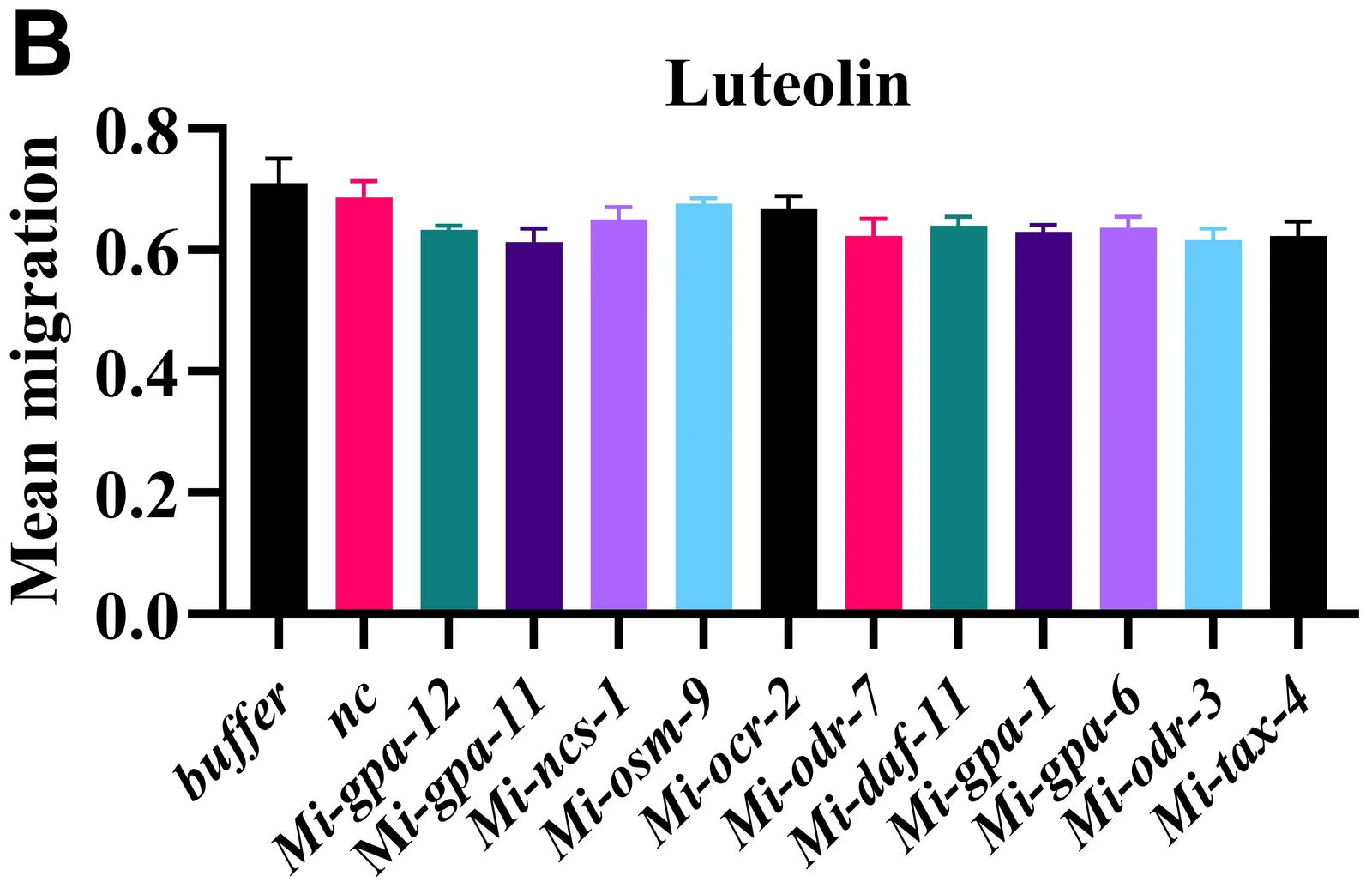

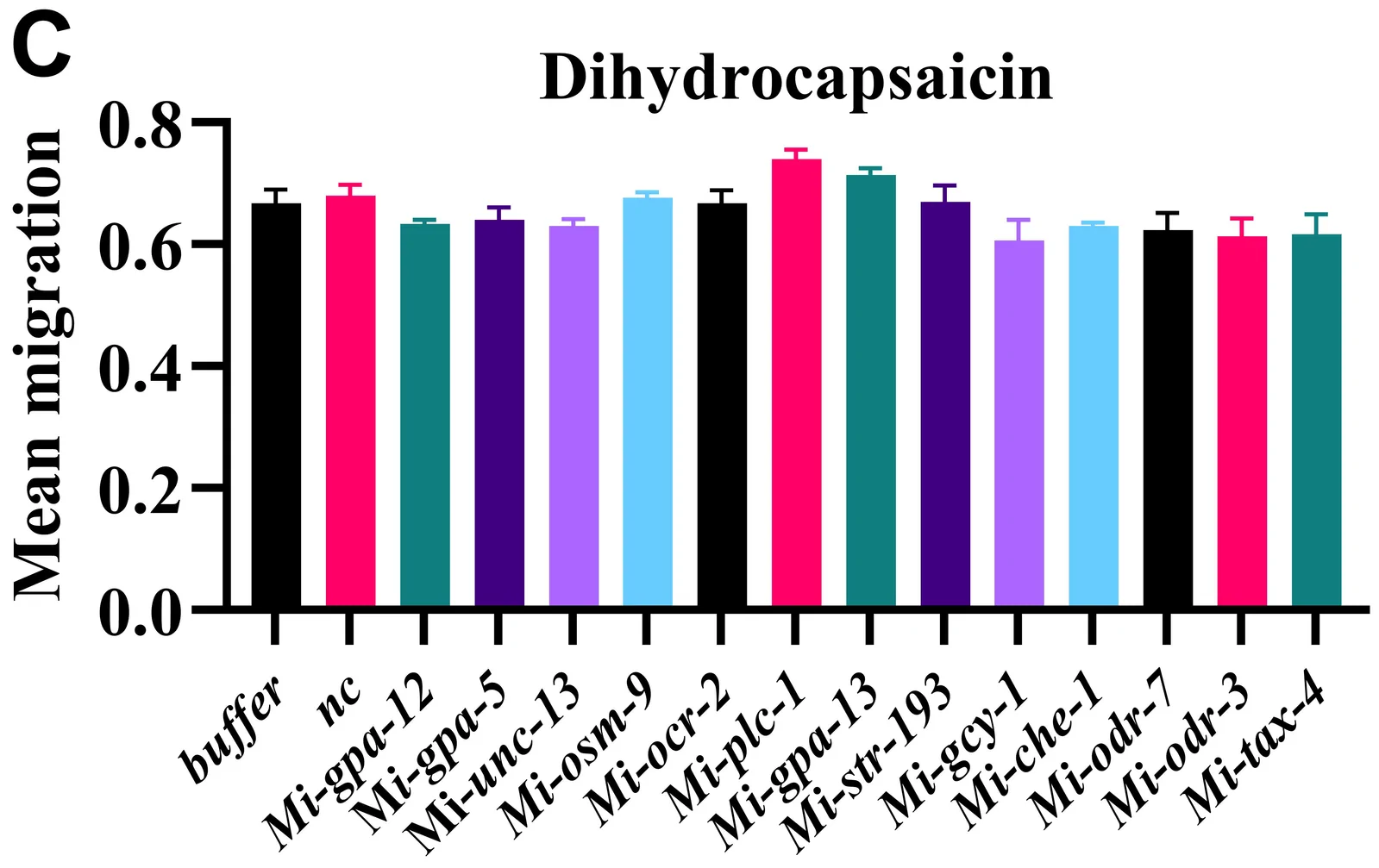

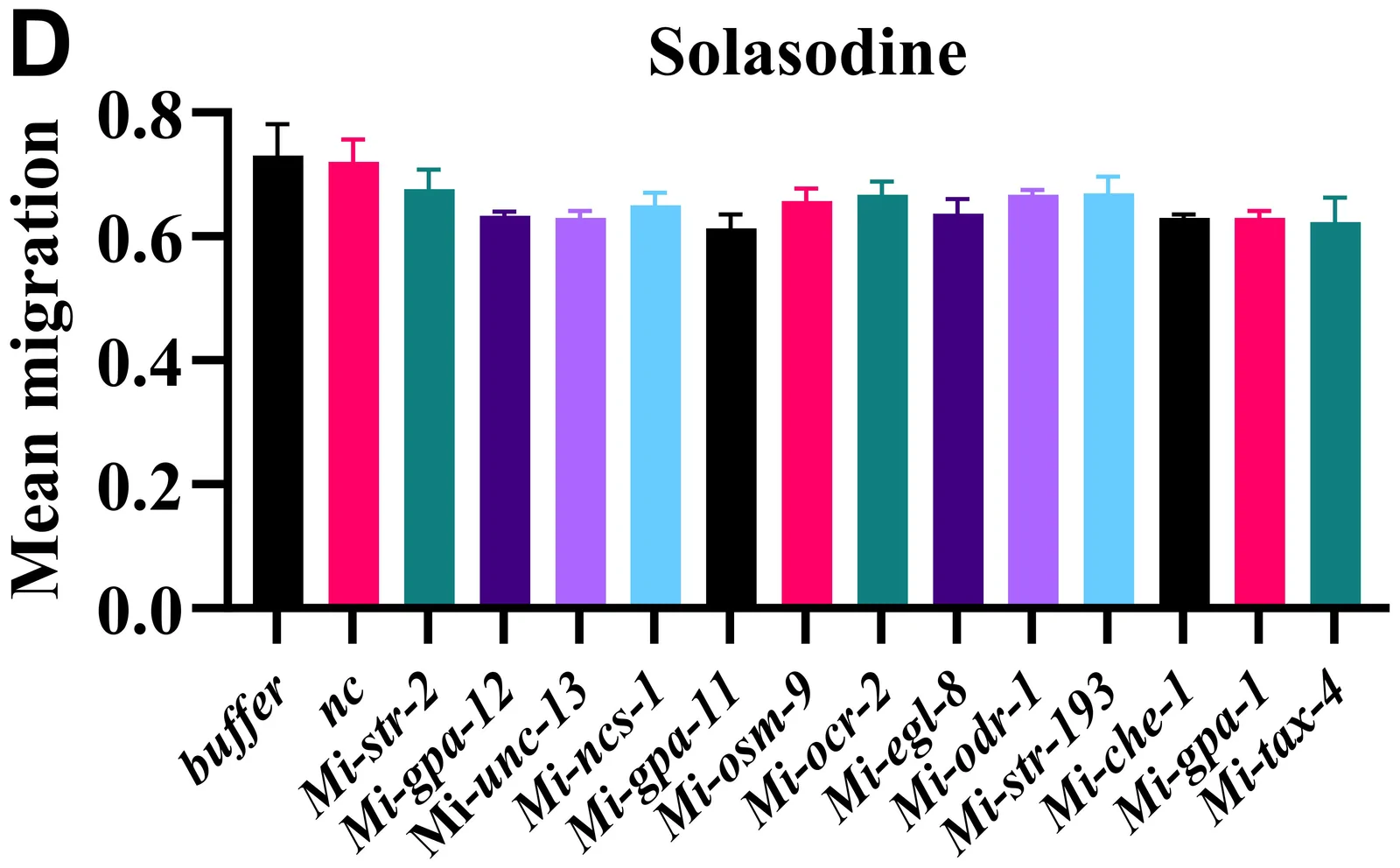
Effects of RNA interference on the migration ability of *M. incognita* J2s. (**A**–**D**) represent migration responses after treatment with quercetin, luteolin, dihydrocapsaicin, and solasodine, respectively. Data are presented as mean ± SD from three independent biological replicates (*n* = 3). Statistical significance was determined using one-way ANOVA followed by Tukey’s multiple comparison test.

The results showed that silencing of different candidate chemosensory genes did not significantly reduce nematode motility compared with the buffer-treated and nc-dsRNA control groups. Similar migration rates were observed among all RNAi-treated groups, including nematodes subjected to gene silencing associated with quercetin, luteolin, dihydrocapsaicin, and solasodine responses, with no significant differences detected among treatments (Figure 5A–D).

These results demonstrated that RNAi-mediated suppression of candidate chemosensory genes did not impair the basal locomotor ability of *M. incognita* J2. Therefore, the altered chemotactic responses following gene silencing were likely associated with changes in chemical signal perception rather than reduced locomotor ability.

### 3.6. RNA Interference Affects Host-Root Chemotaxis and Infectivity of M. incognita

To further investigate the functional roles of candidate chemosensory genes in host localization, tomato root chemotaxis assays and infection assays were performed to evaluate changes in host root recognition and invasion capacity of RNAi-treated *M. incognita* J2.

**Figure 6:**
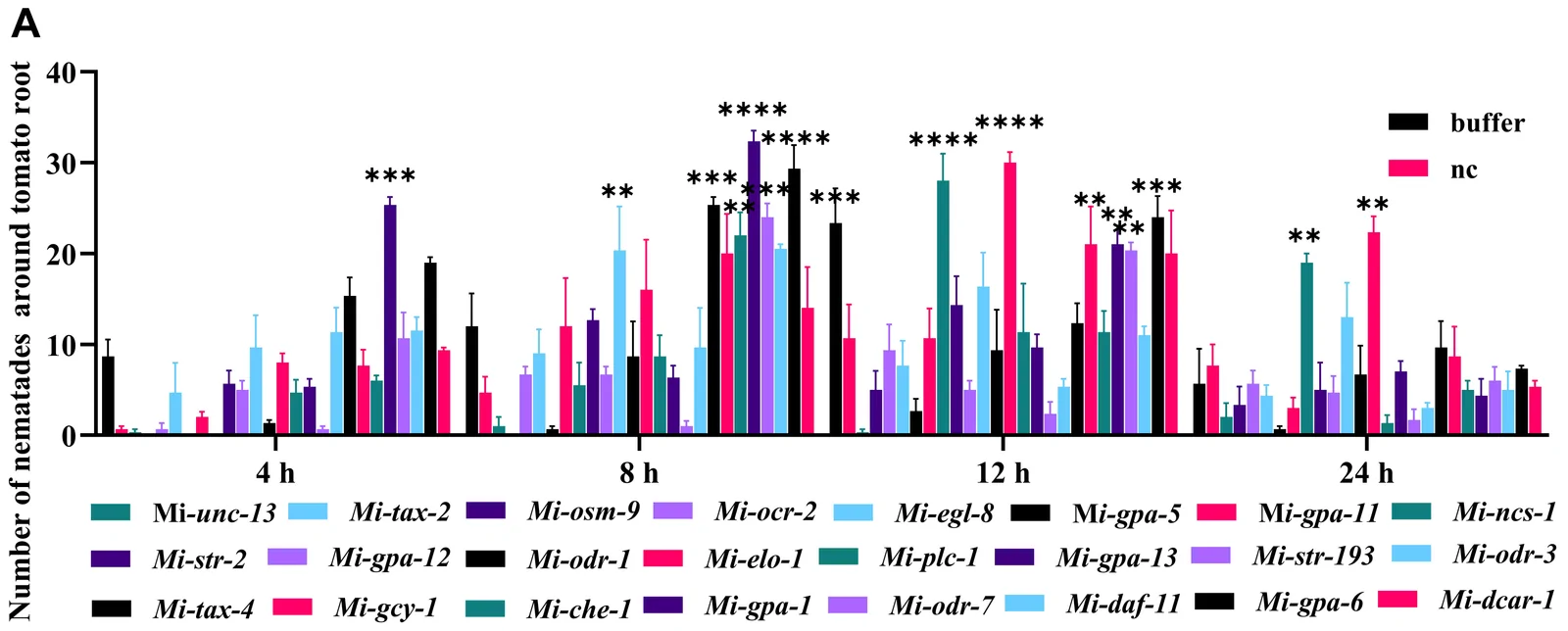

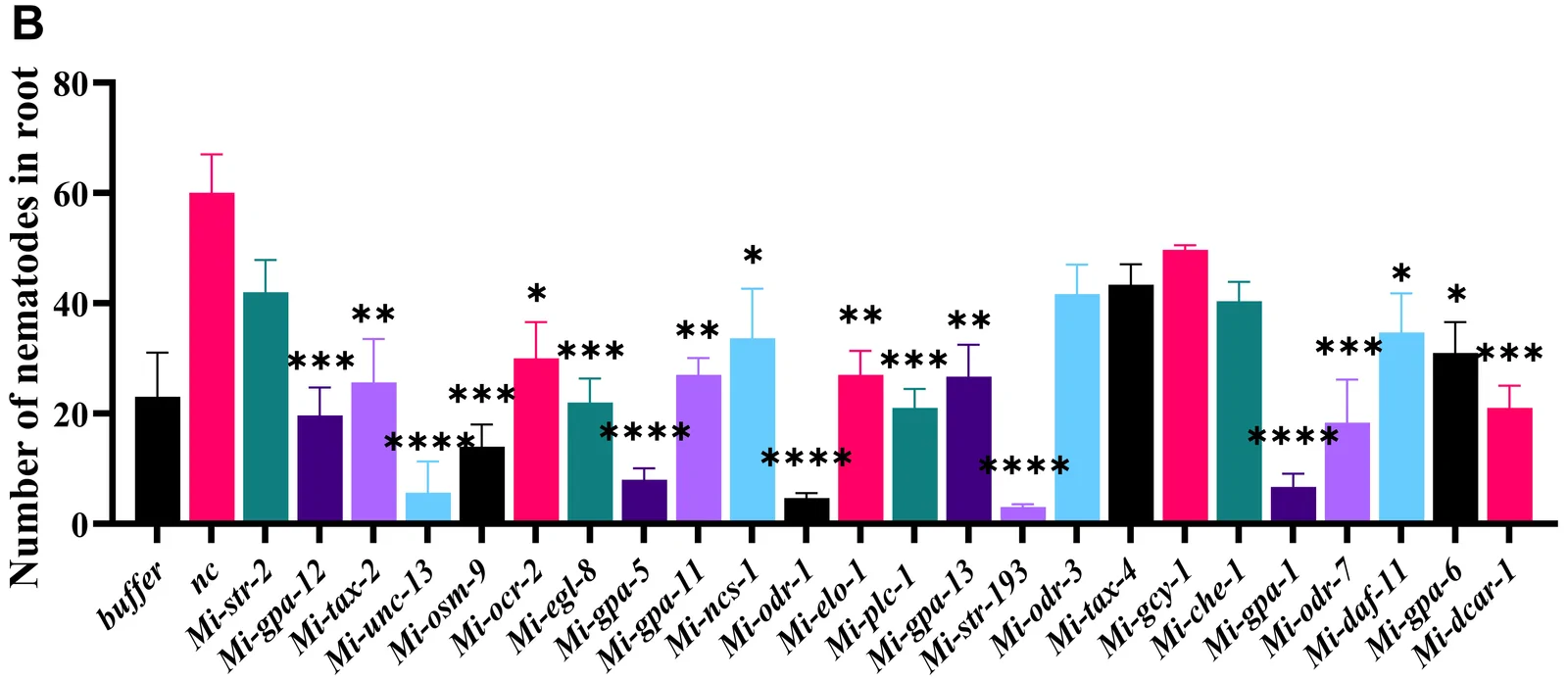
Effects of RNA interference on chemotaxis toward tomato roots and infectivity of *M. incognita* J2s. (**A**) Dynamic migration of RNAi-treated *M. incognita* J2 toward tomato roots after silencing of different candidate chemosensory genes. The number of nematodes accumulated around tomato roots was recorded at 4 h, 8 h, 12 h, and 24 h after inoculation. (**B**) Infection assay of RNAi-treated *M. incognita* on tomato roots. The number of nematodes successfully invading tomato root tissues was quantified after inoculation. Data are presented as mean ± SD from three independent biological replicates (*n* = 3). Statistical significance in (**A**) was determined using two-way ANOVA followed by Tukey’s multiple comparison test, while statistical significance in (**B**) was determined using one-way ANOVA followed by Tukey’s multiple comparison test. Asterisks indicate significant differences compared with the nc-dsRNA-treated group. * *p* < 0.05, ** *p* < 0.01, *** *p* < 0.001, and **** *p* < 0.0001 were considered statistically significant.

First, the effect of candidate gene silencing on host-root chemotaxis was evaluated by dynamically monitoring the accumulation of nematodes around tomato roots after RNAi treatment. Compared with the buffer-treated and nc-dsRNA control groups, silencing of different candidate chemosensory genes resulted in distinct changes in the chemotactic response of *M. incognita* toward tomato roots. At multiple time points (4 h, 8 h, 12 h, and 24 h), several RNAi-treated groups exhibited significantly reduced nematode accumulation around tomato roots, whereas other treatments maintained relatively strong root-directed chemotactic ability (Figure 6A). These results indicated that different chemosensory genes contributed to the recognition of root-derived chemical cues to varying degrees during host localization.

Furthermore, the effects of candidate gene silencing on nematode infectivity were evaluated by quantifying the number of nematodes successfully invading tomato root tissues. Compared with the buffer-treated and nc-dsRNA control groups, RNAi treatment targeting certain chemosensory genes significantly reduced the number of nematodes detected inside tomato roots, whereas other treatments showed limited effects (Figure 6B).

Together, these results demonstrated that RNAi-mediated silencing of candidate chemosensory genes affected both tomato root-directed chemotaxis and infectivity of *M. incognita* to different extents. These findings further indicated that specific chemosensory genes are involved in host localization and infection processes of *M. incognita*.

### 3.7. Construction and Analysis of Candidate Chemosensory Pathways Involved in the Perception of Plant-Derived Chemical Signals

**Figure 7:**
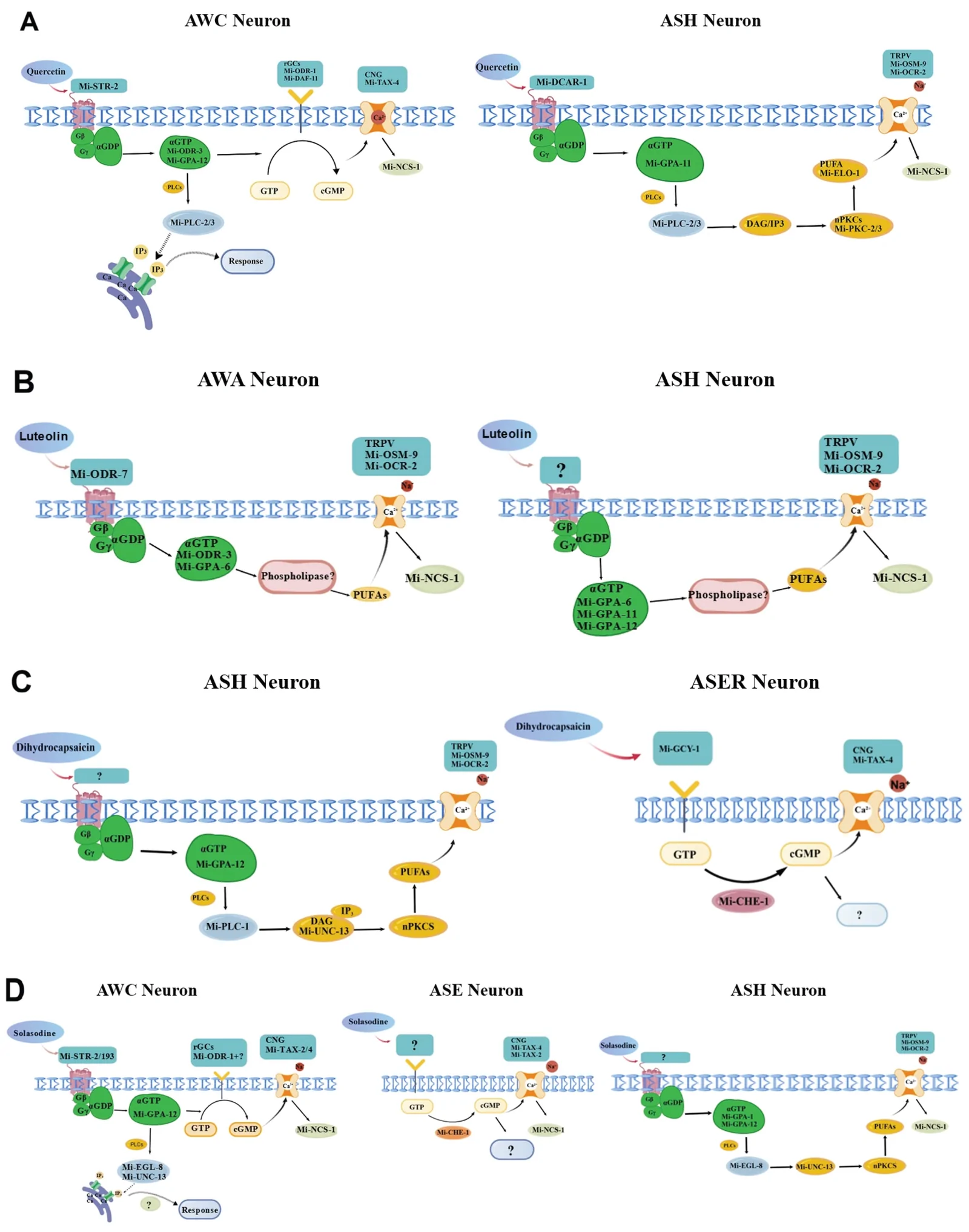
Proposed models of chemosensory pathways involved in the perception of plant-derived chemical signals by *M. incognita*. (**A**) Proposed model for quercetin perception. (**B**) Proposed model for luteolin perception. (**C**) Proposed model for dihydrocapsaicin perception. (**D**) Proposed model for solasodine perception. The predicted interactions among chemical signals, sensory neurons, receptors, G protein signaling components, cyclic nucleotide-gated channels (CNG channels), TRPV channels, and downstream signaling modules represent potential mechanisms that require further experimental verification, the arrows indicate proposed regulatory relationships, and the question marks represent hypothetical interactions that remain to be experimentally validated.

To further explore the potential molecular mechanisms underlying the perception of different plant-derived chemical signals by *M. incognita*, candidate chemosensory pathway models associated with responses to four representative plant-derived compounds were constructed based on the RNAi functional validation results and the previously characterized chemosensory signaling pathways in *C. elegans* (Figure 7).

For the attractant compound quercetin, the predicted perception pathway mainly involved AWC- and ASH-like sensory neurons. In the AWC neuronal pathway, quercetin recognition was predicted to be mediated by the G protein-coupled receptor Mi-STR-2, followed by activation of the downstream G protein signaling cascade and the Mi-ODR-1/Mi-DAF-11-regulated cGMP signaling module. Signal transduction was subsequently mediated through the cyclic nucleotide-gated channel Mi-TAX-4. In the ASH neuronal pathway, quercetin perception was predicted to involve Mi-DCAR-1, with downstream activation of Mi-GPA-11 and the TRPV channels Mi-OSM-9/Mi-OCR-2, leading to ion channel-mediated signal transmission and behavioral responses. These results indicated that quercetin perception in *M. incognita* may be coordinated by multiple sensory neuronal pathways (Figure 7A).

For the attractant flavonoid luteolin, the predicted response pathway differed from that of quercetin. In the AWA neuronal pathway, luteolin perception was mainly associated with the Mi-ODR-7-mediated sensory module, followed by downstream signaling through Mi-ODR-3 and Mi-GPA-6, which subsequently activated the Mi-OCR-2-associated TRPV channel pathway. In the ASH neuronal pathway, luteolin perception involved Mi-GPA-6- and Mi-GPA-11-dependent G protein signaling and further regulated Mi-OCR-2-mediated ion channel activation. These results suggested that structurally related flavonoid compounds could be recognized through distinct sensory signaling modules in *M. incognita* (Figure 7B).

For the repellent compound dihydrocapsaicin, the predicted perception pathway primarily involved ASH- and ASER-like sensory neurons. In ASH neurons, dihydrocapsaicin perception was predicted to activate Mi-GPA-12 through an unidentified upstream receptor, followed by Mi-PLC-1-mediated signal transduction and regulation of the TRPV channels Mi-OSM-9/Mi-OCR-2. In ASER neurons, dihydrocapsaicin recognition was predicted to involve the receptor-type guanylate cyclase Mi-GCY-1 and the Mi-CHE-1-regulated Mi-TAX-4 channel pathway. These results indicated that recognition of repellent compounds by *M. incognita* may require coordinated regulation among multiple sensory neuronal pathways (Figure 7C).

For the repellent alkaloid solasodine, the predicted response pathway involved AWC-, ASE-, and ASH-like sensory neurons. In AWC neurons, solasodine perception was predicted to involve the Mi-STR-2/Mi-STR-193 receptor module, followed by activation of Mi-GPA-12 and regulation of Mi-TAX-4-mediated ion channel signaling. In ASE neurons, the Mi-CHE-1-regulated cyclic nucleotide-gated channel pathway was predicted to participate in solasodine recognition. In addition, Mi-GPA-1 and Mi-GPA-12 in ASH neurons were predicted to contribute to solasodine-induced responses. These findings indicated that solasodine-induced avoidance behavior may be regulated by the integrated activity of multiple chemosensory neurons (Figure 7D).

Collectively, this study established a candidate chemosensory network model underlying the perception of diverse plant-derived chemical signals in *M. incognita* (Figure 7). The model revealed that attractant and repellent compounds were recognized through distinct combinations of sensory neurons and downstream signaling modules. Among these pathways, G protein signaling, cyclic nucleotide-gated channels (CNG channels), and TRPV ion channels appeared to represent central regulatory components involved in chemical signal transduction. These findings provide a molecular framework for understanding how *M. incognita* discriminates complex rhizosphere chemical cues and converts environmental information into host-seeking behavioral decisions.

## 4. Discussion

### 4.1. Plant-Derived Chemical Cues Drive Behavioral Decisions of M. incognita Through Chemosensory Mechanisms

During host infection, second-stage juveniles (J2s) of *M. incognita* rely on environmental chemical information to locate suitable hosts, navigate through the rhizosphere, and initiate root invasion. However, how plant-derived compounds are perceived by root-knot nematodes and subsequently translated into behavioral decisions remains largely unresolved. In this study, we addressed this question by integrating behavioral assays, expression profiling of candidate chemosensory genes, and RNA interference (RNAi)-based functional validation, thereby providing functional evidence linking plant-derived chemical cues, chemosensory genes, chemotactic behavior, and host localization.

Our results demonstrated that plant-derived secondary metabolites with distinct chemical structures induced opposite behavioral responses in *M. incognita*. Consistent with previous observations by Kirwa et al. [9], the flavonoids quercetin and luteolin acted as attractive cues and promoted the directional movement of *M. incognita* J2s. In contrast, the alkaloids dihydrocapsaicin and solasodine elicited strong avoidance responses, supporting the hypothesis proposed by Kuang et al. [13] that specific plant secondary metabolites can function as behavioral regulators for plant-parasitic nematodes. Previous studies have shown that organic acids, amino acids, fatty acids, and secondary metabolites released from plant roots can serve as chemical signals influencing nematode migration and host preference [28]. However, most previous studies have focused on the collective effects of root exudates rather than the specific contribution of individual compounds and their underlying recognition mechanisms. Our findings extend this understanding by demonstrating that plant-derived secondary metabolites are not merely nutritional resources or passive environmental signals, but can act as discrete chemical regulators that directly shape the spatial decision-making process of plant-parasitic nematodes.

An important finding of this study is that structurally similar flavonoids, quercetin and luteolin, although both functioning as attractive signals, triggered distinct transcriptional responses of chemosensory genes in *M. incognita*. This observation indicates that root-knot nematodes do not rely on a single universal sensory pathway for recognizing plant-derived compounds, but instead may employ multiple sensory modules to achieve combinatorial encoding of diverse chemical information. A comparable signal discrimination strategy has been extensively characterized in the model nematode *C. elegans*, where specialized sensory neurons, including AWA, AWC, ASE, and ASH neurons, cooperate with downstream GPCRs, G-protein signaling components, and ion channels to distinguish a broad spectrum of volatile and water-soluble chemical stimuli [29]. Our study provides evidence supporting the possibility that conserved chemosensory principles may also operate in plant-parasitic nematodes, suggesting that *M. incognita* may utilize similar sensory coding strategies to interpret complex chemical landscapes within the rhizosphere. This provides new insights into how plant-parasitic nematodes integrate host-derived chemical information to optimize host-finding behavior.

### 4.2. Chemosensory Genes Mediate Specific Recognition of Diverse Plant-Derived Signals in M. incognita

In nematodes, chemosensory perception generally relies on the coordinated action of sensory receptors, G protein-mediated signal transduction pathways, and downstream ion channels. In the model nematode *C. elegans*, genes such as *osm-9*, *ocr-2*, and *tax-4* participate in signal transduction processes across multiple sensory neurons and regulate chemotaxis, avoidance, and environmental adaptation behaviors [30]. However, whether plant-parasitic nematodes retain comparable chemosensory mechanisms after long-term adaptation to host-dependent lifestyles, and whether different plant-derived signals are recognized through distinct sensory modules, remain largely unexplored. Through integrated analyses of candidate gene expression, RNAi-mediated functional validation, and construction of chemical perception networks, our study demonstrates that different chemosensory-related genes contribute to the perception of plant-derived signals with different chemical properties in *M. incognita*, and suggests the existence of distinct sensory modules, providing a molecular framework for understanding how plant-parasitic nematodes adapt to complex rhizosphere environments.

Although reduced transcript levels are generally expected following RNA interference, inconsistent expression patterns may occur due to off-target effects, compensatory regulation, or feedback mechanisms [31]. Previous studies have demonstrated the existence of transcriptional compensation responses in *C. elegans*, where disruption of specific genes can trigger the activation of related genes to maintain cellular homeostasis [32]. In the present study, several genes, including *Mi-gpa-12*, *Mi-elo-1*, *Mi-gpa-1*, *Mi-gpa-5*, *Mi-gpa-13*, *Mi-tax-2*, and *Mi-egl-8*, exhibited increased transcript abundance after RNAi treatment, whereas corresponding chemotactic behaviors were not significantly altered. These observations suggest that *M. incognita* may compensate for the reduced activity of targeted chemosensory components through transcriptional activation of homologous or functionally related genes, thereby maintaining signal transduction stability. In addition, previous studies have reported transcriptional rebound effects during RNAi in *M. incognita*, potentially associated with feedback regulation induced by intracellular accumulation of small interfering RNAs (siRNAs) [33]. Therefore, these expression changes may reflect an intrinsic regulatory mechanism that preserves neuronal signaling homeostasis in plant-parasitic nematodes.

Our functional analyses revealed that different plant-derived chemical signals induced distinct chemosensory gene response patterns and regulated corresponding behavioral outputs. For example, silencing of *Mi-dcar-1*, *Mi-odr-1*, *Mi-osm-9*, and *Mi-str-2* significantly impaired quercetin-induced attraction, whereas luteolin-mediated attraction relied more strongly on *Mi-odr-7*, *Mi-gpa-6*, and *Mi-tax-4* [34]. These results indicate that chemically similar compounds with identical behavioral outcomes can still be discriminated through different molecular combinations within the nematode sensory system [35]. Similar functional specialization among sensory neurons has been well characterized in *C. elegans*. For instance, AWA neurons are primarily involved in detecting food-associated volatile compounds, whereas AWC neurons mediate responses to a broader range of environmental odor cues; together, these specialized sensory systems enable precise interpretation of complex chemical information [36]. Our findings suggest that *M. incognita* has retained a comparable modular sensory strategy, using different combinations of chemosensory components to distinguish host-derived attractive cues from potentially unfavorable environmental signals.

Beyond signal-specific regulators, we also identified several core components with broader regulatory functions across different chemical responses. For example, silencing of *Mi-osm-9*, *Mi-tax-4*, and *Mi-ncs-1* affected chemotactic responses induced by multiple plant-derived compounds, suggesting that these genes function as common downstream elements involved in general chemical signal transduction. In contrast, *Mi-odr-3* displayed stronger signal specificity, as its silencing markedly affected responses to certain attractive compounds but had limited effects on dihydrocapsaicin-induced avoidance behavior. The coexistence of shared signaling components and signal-specific regulators may provide *M. incognita* with a flexible yet robust strategy for decoding diverse chemical information within heterogeneous rhizosphere environments [37].

Based on these functional validations, we further constructed candidate chemosensory pathway models underlying the perception of different plant-derived compounds (Figure 7). These models suggest that *M. incognita* may employ distinct combinations of chemosensory components to process different plant-derived chemical signals. For example, quercetin perception appeared to involve coordinated regulation through AWC and ASH neurons, whereas luteolin responses were primarily associated with AWA and ASH sensory pathways. Previous work by Li et al. identified genes highly homologous to *C. elegans* AWA- and ASH-associated sensory components in *M. incognita* and demonstrated their importance in tomato infection through RNAi approaches [38]. In *C. elegans*, AWA and AWC neurons detect diverse attractive volatile compounds [29], whereas ASH neurons primarily mediate avoidance responses toward aversive environmental stimuli [39]. Consistent with these functional characteristics, our results suggest that the avoidance responses induced by dihydrocapsaicin and solasodine may be associated with activation of ASH-related sensory pathways, providing a potential behavioral intervention strategy for controlling root-knot nematodes [13]. Collectively, these findings demonstrate that *M. incognita* can selectively recruit distinct sensory networks according to different plant-derived chemical cues, enabling adaptive behavioral decisions in complex soil environments.

Despite the specificity of individual chemical perception pathways, our results also reveal a conserved architecture underlying signal transduction. Multiple plant-derived chemical responses converged on classical chemosensory components, including GPCRs, G protein signaling modules, and TRPV or CNG ion channels. Mombaerts summarized that seven-transmembrane GPCR proteins represent fundamental components of animal olfactory and chemosensory systems and exhibit broad evolutionary conservation among nematodes and other organisms [40]. Furthermore, Liedtke et al. demonstrated that TRPV channels contribute to environmental stimulus perception and play essential roles in sensory regulation in *C. elegans* [41]. Therefore, the “shared core signaling components–specific sensory combinations” model proposed in this study expands the application of established chemosensory concepts from model nematodes to plant-parasitic nematodes and provides a conceptual framework for understanding host recognition mechanisms.

Nevertheless, the chemosensory network proposed in this study still contains several unresolved components. Although the proposed models provide a framework for understanding how plant-derived chemical signals may be perceived by *M. incognita*, they should be interpreted as hypothetical models rather than fully characterized signaling cascades. The experimentally validated evidence in this study is mainly derived from RNAi-mediated functional analyses of candidate chemosensory genes, whereas the predicted connections among specific compounds, sensory neurons, receptors, and downstream signaling components are inferred from homologous mechanisms described in *C. elegans*. In particular, the direct receptors responsible for recognizing specific plant-derived compounds and the downstream roles of signaling molecules such as PLC and PKC require further experimental validation. Future studies integrating sensory neuron localization, single-cell transcriptomic profiling, protein–protein interaction analyses, and genetic complementation approaches will be essential for refining the molecular network underlying plant-derived chemical perception in *M. incognita*. Such investigations will provide deeper mechanistic insights into nematode host recognition and may facilitate the development of behavior-based strategies for sustainable root-knot nematode management.

### 4.3. Chemosensory Regulation Connects Plant-Derived Signal Perception with Host Localization and Infection Processes

For plant-parasitic nematodes, chemosensation not only determines behavioral responses toward environmental cues but also directly influences their ability to successfully locate and infect suitable hosts. Therefore, elucidating the role of chemosensory genes in host localization provides critical insights into the molecular mechanisms underlying parasitic adaptation in root-knot nematodes.

In this study, silencing of candidate chemosensory genes significantly impaired the chemotactic localization of *M. incognita* toward tomato roots and subsequently reduced its infectivity, whereas RNAi treatment did not affect the basic locomotory ability of J2s. This result excludes the possibility that reduced chemotactic responses were caused by general movement defects and indicates that the observed behavioral alterations primarily resulted from disrupted chemical perception and decision-making processes. Previous studies have demonstrated that *M. incognita* J2s can perceive diverse chemical cues released by plant roots and utilize this information to establish directional migration patterns, ultimately facilitating root-tip localization and successful infection establishment [29]. However, the molecular regulators responsible for linking chemical perception with host-finding behavior remain poorly understood.

Our findings further demonstrate that several plant-derived chemical signal-responsive genes are not only involved in the recognition of individual compounds but also contribute to host root perception. Following silencing of specific chemosensory genes, fewer nematodes accumulated around tomato roots, accompanied by a reduction in the number of nematodes successfully entering root tissues. These results reveal a continuous regulatory relationship between plant-derived signal perception and host infection, in which nematodes first decode environmental chemical information through chemosensory systems and subsequently regulate directional movement to achieve host localization. Thus, the chemosensory machinery of *M. incognita* represents a critical molecular interface connecting environmental perception with parasitic success.

### 4.4. Limitations and Future Perspectives

Although this study reveals important molecular mechanisms by which plant-derived chemical signals regulate chemotactic behaviors in *M. incognita*, several limitations remain to be addressed.

First, the behavioral analyses in this study were primarily performed using agarose plate-based chemotaxis assays to evaluate responses toward individual chemical compounds. Wang et al. reviewed recent advances in the use of Pluronic gel-based systems for simulating three-dimensional soil environments in plant-parasitic nematode chemotaxis studies and highlighted the limitations of conventional assays in reproducing complex chemical gradients present in natural rhizospheres [42]. Under field conditions, *M. incognita* encounters heterogeneous chemical landscapes generated by the combined release of numerous root exudate components rather than isolated compounds. Therefore, future studies integrating soil microenvironment simulation, multi-component chemical gradient analysis, and field-scale validation will be necessary to determine the ecological relevance of these chemical cues during natural host-finding processes.

Second, although similar to the study by Kamaraju et al., which demonstrated that host-induced RNAi targeting the neural regulatory gene *Mi-flp14* can interfere with parasitic success and highlighted the potential of RNAi for dissecting nematode neuroregulatory networks and developing control strategies [43], the downstream neural circuits underlying plant-derived chemical perception remain incompletely characterized. Future investigations combining single-cell transcriptomic analyses, sensory neuron mapping, and genetic complementation approaches will be essential for establishing a more comprehensive regulatory network linking chemical perception to behavioral output and infection development.

Collectively, by integrating behavioral analyses, RNAi-mediated functional validation, and chemosensory pathway modeling, this study uncovers a previously underappreciated mechanism through which plant-derived secondary metabolites regulate behavioral decisions in *M. incognita*. We propose a regulatory model consisting of “plant chemical signals–chemosensory pathways–chemotactic behavior–host infection”, which provides a mechanistic framework for understanding chemical communication between plants and parasitic nematodes. Beyond expanding current knowledge of plant–nematode chemical interactions, this model offers new perspectives for deciphering neuronal sensory networks in plant-parasitic nematodes and provides a theoretical foundation for developing sustainable control strategies based on disruption of host-recognition processes.

## Data Availability

The original contributions presented in this study are included in the article/Supplementary Material. Further inquiries can be directed to the corresponding author.

## Supplementary Materials

The following supporting information can be downloaded at https://www.mdpi.com/article/10.3390/pathogens15080860/s1: Table S1: Homology relationships between chemosensory-related genes of *Meloidogyne incognita* and Caenorhabditis elegans; Table S2: Primer sequences used for RT-qPCR analysis in *Meloidogyne incognita*; Table S3: Primers used for dsRNA synthesis with T7 promoter sequences in *Meloidogyne incognita*.
