# Supplementary material for: Chemosensory Pathways Involved in the Perception of Tomato-Derived Flavonoids and Alkaloids in Meloidogyne incognita
Source: Pathogens. 2026 Aug 19;15(8):860. doi: 10.3390/pathogens15080860 (PMC13516830; doi:10.3390/pathogens15080860)
Supplement: Supplementary file 1 [file pathogens-15-00860-s001.zip › pathogens-4487528-supplementary.pdf]

Supplementary Materials

Chemosensory pathways involved in the perception of tomato-derived flavonoids and alkaloids in *Meloidogyne incognita*

Qian Wang, Liping Peng, Youjing Wang, Jiajia Hu, Liangying Kong, Yajun Liu*

State Key Laboratory for Conservation and Utilization of Biological Resources in Yunnan Province, Kunming 650032, China * Correspondence: lyj@ynu.edu.cn; Tel.: +86-871-65031093

Table S1

Homology relationships between chemosensory-related genes of *Meloidogyne incognita* and *Caenorhabditis elegans*.

M.incognita	***C.elegans***
Minc3s00050g02698	*odr-7*
Minc3s01623g25167	*odr-10*
Minc3s02801g31617	*str-2*
Minc3s00015g01050	*odr-3*
Minc3s00008g00543	*dcar-1*
Minc3s01507g24354	*che-1*
Minc3s10403g44051	*gcy-1*
Minc3s00047g02554	*ocr-2*
Minc3s00048g02626	*gcy-8*
Minc3s00270g09074	*unc-13*
Minc3s02293g29297	*gcy-11*
Minc3s06981g40576	*str-193*
Minc3s00489g13190	*tax-4*
Minc3s00376g11244	*gpa-2*
Minc3s00022g01386	*gpa-5*
Minc3s01972g27520	*gpa-6*
Minc3s00003g00191	*gpa-11*
Minc3s01954g27399	*gpa-12*
Minc3s00015g01050	*gpa-13*
Minc3s00056g02910	*odr-1*
Minc3s00726g16570	*daf-11*
Minc3s05559g38432	*egl-8*
Minc3s00483g13114	*elo-1*
Minc3s00012g00794	*plc-1*
Minc3s01282g22437	*plc-2*
Minc3s04427g36263	*plc-3*
Minc3s00696g16213	*pkc-1*
Minc3s00177g06844	*pkc-2*
Minc3s00167g06595	*pkc-3*
Minc3s00025g01614	*gcy-9*
Minc3s00057g02942	*osm-9*
Minc3s02630g30946	*gcy-4*
Minc3s00096g18287	*tax-2*
Minc3s00187g07094	*gpa-1*
Minc3s01909g27122	*ncs-1*

Table S2

Primer sequences used for RT-qPCR analysis in *Meloidogyne incognita*.

Genes	Prime F (5'-3')	Prime R (5'-3')
Mi-tax-4	ACGCTGAGGAAAGTTAGGCT	TCTCTCTTCCAACATCGCCTT
Mi-tax-2	TTGTAGAAAAGGGGATGTTGGT	GCTGTTCTACGATTATTTCCGC
Mi-osm-9	TTACGTCCTGTGGAAACCGA	TGCTTTGAGTGCGTAGTTCG
Mi-ocr-2	AGAAATGAACATTTGGGAGCTT	TTCCACCTTTGCCTCCATCT
Mi-odr-1	ACACTTTTCGTTCTGCCTTCC	TTGGTCAATTTCAACACCCAATT
Mi-daf-11	ACACTGAAAATGCCTGGAAGT	GTTCATCAACCACCGACTTTG
Mi-gpa-2	TCTATTCGAGTTTGTTTCAAGGA	TGTCTGTAGCACATGTTTGGTG
Mi-gpa-5	AGTCCAACAGAAGCGGATGT	TGACCGCCAACATCAATGAG
Mi-gpa-6	GGAATTTGTTCACGCCTCCA	GCTGTAAAGCCTGACTCGTG
Mi-gpa-11	CGCATAACATGACCGAGGTG	TGCAGCCTTCTCCATACTGA
Mi-gpa-12	CCGAGATGAGATGGCTGCTA	CTTTACCGCTGCAAAGACGA
Mi-gpa-13	ACCTGGCTATAGACCCACAG	TTCCGAACGTTGTCCACCTA
Mi-dcar-1	TCAGCAAAGTATGGTCTGACA	CCCAATTGCTCTATTCACCA
Mi-plc-2	AGAGCTGTGAGAATGAAGAGGT	ATCGAGTGATTTGCAGACAA
Mi-plc-3	TGCCTTTCGAACTAGCGATT	AAGGGCAGAAGGTGATGGAA
Mi-pkc-2	GTCTGCACATTGGTTGTCCA	CCATCTGTGTTGCTGTCTGG
Mi-pkc-3	GGAGCATGGGTTTGGATGAC	ATCCAGTCGGTGTCCTCTTC
Mi-odr-7	GCTGCTTCAAATGGCCTTCA	TCCTTGCGTTCCAATATCGC
Mi-plc-1	TAGGCGTGTTAAGGGAAGCA	ATTGAAGGTGGACGGACCAT
Mi-pkc-1	CACAACGCCGTTTAGGATGT	GCATCCCAGTCGATTTCTCG
Mi-ncs-1	ACTCGGTCAGATTATCCGATG	AGGGCATGTACAATACTGGGA
Mi-egl-8	TGCTGCTCATTGTTCTGTTAGG	ATAAGCTGTTGGCAGGGACT
Mi-unc-13	AAGGAAGTGGAGGAGGAGGA	AATTATCACGTGCCGCATCC
Mi-elo-1	ACAGAGTTCGTATTGTCATGTCC	AGTAGTGGCCGTTTCCTCAA
Mi-che-1	ACAGCTCACAGACGAGTACA	ATGTGGGCAGGGATAAGGTC
Mi-gcy-1	GGGTTGATTGCCCTTCACAG	CGCTAATCTTTACTTGCCAACG
Mi-gcy-4	TGCCAAGGCAATTGCTGAA	CGAGGCATTGTCAATCCAACA
Mi-gcy-8	TTGGCTGCCAGAATTGCTTT	TTACCTGTCCCGAAGAGACG
Mi-gcy-9	TGGCAGACTTGTCTCTCGTT	CGAGCCATTTCTGTTGCTGA
Mi-gcy-11	GCTGACTTCATGGCTCATCC	TTCTCTCCGTCGAAACCACA
Mi-gcy-14	TACTGGGACGGAATGTCCAC	GACTGCCTCTTTGGCAATGT
Mi-odr-3	ACCTGGCTATAGACCCACAG	TTCCGAACGTTGTCCACCTA
Mi-str-193	CCATTGCTCTAAGTTGGGTTGT	AGCCTGAATGAGAAGAGTCCT
Mi-str-2	GTCGTTTATTGGGGCTGCAA	ACGAAATAGATAAACGGCCCA
β-actin	CGCAATCTTGCGTCTTGACT	CACGGACATCTCACGTTCGG

Table S3

Primers used for dsRNA synthesis with T7 promoter sequences in *Meloidogyne incognita*.

Genes	Prime F (5'-3')	Prime R (5'-3')
Mi-tax-4	TAATACGACTCACTATAGGGAGT CCCGTCGATACCTCTACAGT	TAATACGACTCACTATAGGGAGAAAG TCTCCCGGGCTGAAAA
Mi-tax-2	TAATACGACTCACTATAGGGAGG CGATGTTGGAAGAGAGATG	TAATACGACTCACTATAGGGAGCTGC TGCAATCGAGACAAT
Mi-osm-9	TAATACGACTCACTATAGGGAGA ACGAAGCAAAATCCGCCTT	TAATACGACTCACTATAGGGAGAGCC TTCCATTTGTCAGCT
Mi-odr-1	TAATACGACTCACTATAGGGAGT TTGAGTAAATTCCCGCCGG	TAATACGACTCACTATAGGGAGGAT CGCCATTCATTGAGCCA
Mi-unc-13	TAATACGACTCACTATAGGGAGT ACAAAGAGCCGCCGAAAAG	TAATACGACTCACTATAGGGAGCGAA TTGCTCCGCTAACAACT
Mi-ocr-2	TAATACGACTCACTATAGGGAGG GAGGGCCAATACATACAGAA	TAATACGACTCACTATAGGGAGCCTC ACTTTCCCCATAAACAACT
Mi-gpa-1	TAATACGACTCACTATAGGGAGT ACTTGGTGCAGGGGAAAGTG	TAATACGACTCACTATAGGGAGCCCC AACATCAAATACTCTCCA
Mi-gpa-5	TAATACGACTCACTATAGGGAG AGCCGAATCAAGCAGAATTCT	TAATACGACTCACTATAGGGAGTG CTTTCTGGCTCCTTCACA
Mi-gpa-11	TAATACGACTCACTATAGGGAGC GGAGGGCACGTACTATAGA	TAATACGACTCACTATAGGGAGCGG AGGGCACGTACTATAGA
Mi-gpa-12	TAATACGACTCACTATAGGGAGT GCCAACGGAACAAGACATT	TAATACGACTCACTATAGGGAGTA CCGCTGCAAAGACGAATC
Mi-gpa-13	TAATACGACTCACTATAGGGAGT ATTGTTGGGGGCAGGAGAG	TAATACGACTCACTATAGGGAGCC GAACGTTGTCCACCTACA
Mi-odr-7	TAATACGACTCACTATAGGGAGA GTAGAACCAGCAGTTCTGTC	TAATACGACTCACTATAGGGAGGAA AAGCGTCTCTGGAAGCAT
Mi-gpa-6	TAATACGACTCACTATAGGGAGG CGATTGTGGACTGATTCTGG	TAATACGACTCACTATAGGGAGCAT CTGTTACGGCATCAAATACA

Mi-plc-1	TAATACGACTCACTATAGGGAGAAGAGCATGAACCCGATCCT	TAATACGACTCACTATAGGGAGGAGAGGGCAATCTAGGGCTC
Mi-ncs-1	TAATACGACTCACTATAGGGAGGTGCTGATATGGCCAAACCTC	TAATACGACTCACTATAGGGAGCCACTCGCTTTTCAGGGCTA
Mi-egl-8	TAATACGACTCACTATAGGGAGTTTTATGCGACGTCCAGACC	TAATACGACTCACTATAGGGAGTGCGCAGAAAGGTAAGCTTT
Mi-dcar-1	TAATACGACTCACTATAGGGAGTAAAGCCCACAGAGTCAGCAG	TAATACGACTCACTATAGGGAGACCGTTTCCGAACACTTCTTTG
Mi-elo-1	TAATACGACTCACTATAGGGAGTCTCGCCATTTTCTCAACCA	TAATACGACTCACTATAGGGAGAGCAGACCCATTACTAGCCT
Mi-che-1	TAATACGACTCACTATAGGGAGTGGCATATGGGAGGAAGTGG	TAATACGACTCACTATAGGGAGGCTAAATGGCTTCTCACCTGT
Mi-gcy-1	TAATACGACTCACTATAGGGAGTGGTTAGGGATGGGAGTCAA	TAATACGACTCACTATAGGGAGCGAGGCATTGTCAATCCAACA
Mi-odr-3	TAATACGACTCACTATAGGGAGGACAAGTGATAGACGTGCTGC	TAATACGACTCACTATAGGGAGTGACTTCCACAACGCCTGTA
Mi-str-193	TAATACGACTCACTATAGGGAGCCATTGCTCTAAGTTGGGTTGT	TAATACGACTCACTATAGGGAGTTTAAACGTGGTGGTGCTGT
Mi-str-2	TAATACGACTCACTATAGGGAGGAGAGATAAACCAAGCCAGTGC	TAATACGACTCACTATAGGGAGGCGTCAGGATACCCAGCATA

Note: The sequence TAATACGACTCACTATAGGGAG corresponds to the T7 promoter sequence.
